# Geospatial analysis reveals similar HIV-1 drug resistance patterns among antiretroviral therapy-experienced children and adolescents (0-17 years) of South Africa

**DOI:** 10.1038/s43856-026-01770-9

**Published:** 2026-07-07

**Authors:** Lilishia Gounder, Moherndran Archary, Aabida Khan, Kerusha Govender, Raveen Parboosing, Kogieleum Naidoo, Benjamin Chimukangara

**Affiliations:** 1 Department of Virology, School of Medicine, University of KwaZulu-Natal, Durban, South Africa; 2 Department of Virology, National Health Laboratory Service, Inkosi Albert Luthuli Central Hospital, Durban, South Africa; 3 Department of Paediatrics, University of KwaZulu-Natal, Durban, South Africa; 4 School of Pathology, University of Witwatersrand & National Health Laboratory Service, Johannesburg, South Africa; 5 Centre for the AIDS Programme of Research in South Africa (CAPRISA), Durban, South Africa; 6 South African Medical Research Council (SAMRC), CAPRISA HIV-TB Pathogenesis and Treatment Research Unit, Durban, South Africa; 7 Critical Care Medicine Department, NIH Clinical Center, Bethesda, MD USA

**Keywords:** HIV infections, Paediatric research

## Abstract

**Background:**

HIV-1 drug resistance (HIVDR) complicates antiretroviral therapy (ART) in children and adolescents living with HIV (CALWH), particularly in high-prevalence areas such as KwaZulu-Natal, South Africa. This study describes trends in HIVDR and patterns by drug class and geographic location among ART-experienced CALWH in KwaZulu-Natal.

**Methods:**

We analysed 914 routine HIV-1 genotypic resistance tests (GRTs) obtained between January 2018 and June 2022 from 0–17-year-old CALWH, integrating facility-linked Geographic Information Systems (GIS) coordinates for geospatial, descriptive and logistic regression analyses.

**Results:**

Here we show that 80.96% (740/914) of GRTs from CALWH (median age 13, interquartile range 7-15 years) across 114 facilities demonstrate HIVDR. Of the 740 individuals with HIVDR, 609 (82.30%) had non-nucleoside reverse transcriptase inhibitor (NNRTI) mutations, and among them, 32 (5.25%) were on NNRTI treatment. Additionally, 525 (70.95%) had nucleoside reverse transcriptase inhibitor (NRTI) mutations, and 181 (24.46%) had major protease inhibitor (PI) mutations. One in every 6 GRTs with HIVDR (129/740; 17.43%) have triple-class NRTI, NNRTI and PI mutations. When analysed by age, ~9 in every 10 GRTs from CALWH aged 0-9 years (237/257, 92.22%) have HIVDR, compared to 76.56% (503/657) aged 10-17 years (OR = 3.63, 95% CI: 2.22-5.93, p < 0.001). GIS-interpolation maps show potential-low-level resistance to tenofovir, low-to-intermediate-level resistance to abacavir (high-level in northern localities) and intermediate-to-high-level resistance to lamivudine, nevirapine and efavirenz.

**Conclusions:**

Routine GRTs demonstrate high HIVDR prevalence among younger children, while ART susceptibility maps show overall uniformity with variations in certain regions, such as northern KwaZulu-Natal localities. GIS-linked real-time monitoring of HIVDR is crucial to assess the impact of the revised HIV-1 treatment guidelines for CALWH in South Africa.

## Plain language summary

HIV-1 drug resistance (HIVDR) is a phenomenon that makes the standard antiretroviral therapy (ART) treatment for HIV-1 less effective, posing a challenge to HIV-1 treatment programmes worldwide. Our study describes how HIVDR has changed over time among children and adolescents living with HIV (CALWH) in KwaZulu-Natal province, South Africa. We find that children failing ART aged 0-9 years have higher HIVDR prevalence compared to those aged 10-17 years. We also analyse geography to link HIVDR data to healthcare facility locations. We note higher levels of HIVDR to certain drugs across the province, with high-level resistance in northern KwaZulu-Natal. This study highlights the urgent need for geographically-linked real-time monitoring of HIVDR in high prevalence settings for CALWH with ART failure.

## Abstract (web summary)

Gounder et al. describe trends of HIV-1 drug resistance (HIVDR) among children and adolescents living with HIV in KwaZulu-Natal (KZN), South Africa. Routine HIV-1 genotypic resistance tests integrated with geospatial analyses show higher HIVDR prevalence among younger children and overall uniformity in antiretroviral drug susceptibility across KZN.

## Introduction

Despite the progress made in antiretroviral therapy (ART) and the improvements in therapeutic outcomes for people living with HIV, HIV-1 drug resistance (HIVDR) constitutes a significant global challenge to sustained lifelong therapy^1^. Children and adolescents living with HIV (CALWH) in resource-limited settings face an increased risk of developing HIVDR. This is due to limited child-friendly drug formulations, adherence challenges, dose adjustments, maternally transmitted HIVDR and potential prior exposure to non-nucleoside reverse transcriptase inhibitor (NNRTI)–based ART for prevention of vertical transmission (PVT)^2–5^. In 2013, the World Health Organization (WHO) recommended ritonavir-boosted lopinavir (LPV/r)-based ART as the preferred regimen for CALWH aged <3 years, regardless of PVT drug exposure history^6^.

South Africa, home to the largest pediatric HIV-1 epidemic globally^7^, implemented guidelines in 2013 that recommended abacavir (ABC), lamivudine (3TC), and LPV/r as the first-line ART regimen for children aged 0–3 years, with efavirenz (EFV) replacing LPV/r for CALWH > 3 years of age^8^. Progressively, tenofovir disoproxil fumarate (TDF) replaced ABC in treatment regimens for CALWH aged >10 years and ≥30 kg, while all NNRTI-based regimens were transitioned to LPV/r or dolutegravir (DTG) prior to and during the rollout of DTG in December 2019^3,9^. Subsequently, WHO and South African national guidelines recommended DTG-based regimens in all children >1 month of age and ≥3 kg, with current first-line regimens: ABC + 3TC + DTG (<30 kg) or TDF + 3TC + DTG (≥30 kg), and protease inhibitors (PIs) reserved for individual cases where DTG is unsuitable^10,11^. Despite the availability of more robust regimens in ART programs, progress towards the UNAIDS 95-95-95 targets for CALWH remains stagnant, with only 63% of eligible children aged 0–14 years receiving ART in South Africa, and just 54% of treated children from Eastern and Southern Africa attaining virological suppression in 2023^12–14^.

Viral load (VL) non-suppression among CALWH is closely linked to suboptimal adherence, a major factor in treatment failure and the emergence of HIVDR^15–17^. A systematic review conducted in 2024, which included 28539 CALWH from 52 countries, found that the global prevalence of HIVDR was 74.16% among ART-experienced children and 26.31% among ART-naïve children, with the highest HIVDR prevalence of 37.80% [95% confidence interval (CI) 26.24–50.08] observed among ART-naïve children in Southern Africa^16^. South Africa is home to approximately 10% of the global population of CALWH^13^. Despite this, pretreatment genotypic resistance tests (GRTs) are not routinely recommended for CALWH in national guidelines, except in cases where the child is perinatally infected, and the mother has failed a ritonavir-boosted protease-inhibitor (PI/r)- or DTG-based regimen^11^.

Given the high prevalence of HIVDR among pediatrics in South Africa^3^, there is a critical need for effective near-real-time surveillance of HIVDR in CALWH. Geospatial analysis has the potential to be an effective surveillance technique for predicting, via interpolation approaches, areas with increased HIVDR in resource-limited settings. Beyond descriptive mapping, geospatial analysis of antiretroviral (ARV) drug susceptibility levels can reveal localities of high-level resistance, even within regions where HIVDR appears uniformly distributed. Such geospatial analyses promote targeted and optimized changes to existing treatment programs, enabling timeous test requests for linked ARV drug levels and GRTs, providing earlier detection of HIVDR in CALWH. Combining geospatial analyses with health statistics, mobile network data, and environmental data allows for the prediction of HIV burden estimates and new infections, with the future possibility of detecting at-risk geospatial localities ahead of traditional surveillance systems^18,19^. Further integration of the geospatial-health statistics model with machine learning and explainable artificial intelligence may enable, for example, the identification of appropriate candidates for pre-exposure prophylaxis within a defined hyperendemic region or uncover disease determinants of infectious diseases across a specific region^18,19^. Ultimately, utilizing these geo-localized analyses, policymakers can make more informed decisions regarding the allocation of healthcare workers to rural versus urban primary healthcare facilities or enhance specialized infectious disease services in regions with sustained high-level viremia and HIVDR among treatment-experienced CALWH.

In this study, we combine data from routine GRT records with Geographic Information Systems (GIS) data to survey and investigate the geospatial variations of HIVDR among CALWH in the hyperendemic province of KwaZulu-Natal (KZN), in South Africa. We use interpolation maps to demonstrate ARV drug susceptibility levels across healthcare facilities within the 11 districts of KZN province. We also analyze HIVDR mutations by drug class and predict genotypic susceptibility scores (GSSs) to determine whether the existing drug regimens remain effective for CALWH from varying age categories. Overall, our findings offer a geospatial perspective of the HIVDR burden among CALWH in KZN and underscore its importance in HIVDR surveillance.

## Materials and methods

### Study population and design

This retrospective cross-sectional observational study analyzed routine HIV-1 GRT records from the National Health Laboratory Service (NHLS) Central Data Warehouse (CDW), utilizing secondary data with GIS information spanning the period from 1 January 2018 to 30 June 2022. The study period encompasses the pre-transition and transition phases to DTG as part of first-line ART regimens in South Africa, aligning with our previous research on geospatial mapping of HIV-1 VLs^20^ and HIVDR among individuals >17 years of age^21^. The NHLS is South Africa’s largest provider of diagnostic laboratory services, serving ~80% of the population, and playing a critical role in supporting the Department of Health’s oversight of national and provincial programs^22^. The South African programmatic indication for GRTs is virological failure, defined as two or more VLs ≥1000 copies/mL of plasma taken ≥2 years after initiating DTG- or PI/r-based regimens, with >80% patient adherence to ART^11^. However, previous guidelines published in 2015 and 2020 recommended GRTs after consultation with a specialist for CALWH with increased VLs on PI- or DTG-based regimens (regardless of treatment duration) with concurrent rifampicin-containing TB therapy. GRTs were also recommended for newly diagnosed children <2 years of age whose mother received PI-based ART during pregnancy or lactation^5,9^. Essentially, GRT is indicated in specific scenarios of virological failure according to the standard of care in South Africa. In this study, viral RNA extracted from patient plasma specimens with VLs ≥1000 copies/mL was genotyped and analyzed for the HIV-1 *protease* (PR), *reverse transcriptase* (RT) and *integrase* (IN) genes, as described previously^21^.

### HIV-1 genotypic data analysis

We analyzed HIV-1 genotypic data from all children (0–9 years of age) and adolescents (10–17 years of age)^10^, who received healthcare at public-sector healthcare facilities (*n* = 114) in KZN. We excluded GRT records obtained from individuals ≥18 years of age (i.e., adults). We used data from individuals <18 years to develop an interactive database for geospatial analysis and data visualization. We identified HIVDR mutations and assessed ARV drug susceptibilities using the Stanford University HIV Drug Resistance Database v9.6^23^. We assessed drug susceptibilities for nucleoside/nucleotide reverse transcriptase inhibitors (NRTIs), NNRTIs and PIs, and defined HIVDR as any GRT showing reduced drug susceptibility (i.e., potential low-level resistance to high-level resistance) to any ARV drug available in standard of care regimens in South Africa^21^. Given that only a few GRTs (*n* = 8) included IN genotyping, and to avoid bias, we did not conduct geospatial analysis of the integrase strand transfer inhibitor (INSTI)-resistance mutations.

### HIVDR database curation and genotypic susceptibility scores

Using the GRT records, we created an HIVDR database categorized according to the absence or presence of HIVDR, i.e., decreased susceptibility to ≥1 ARV drug. We included the following metadata for each GRT record: patient age, sex, date and location of specimen collection (i.e., healthcare facility name, district and subdistrict), patient status (i.e., outpatient or inpatient/admission), most recent VL, current ART regimen, HIVDR mutations detected and ARV drug susceptibility. We further categorized age into four groups: (i) 0–4 years, (ii) 5–9 years, (iii) 10–14 years, and (iv) 15–17 years, relative to the regimens that were used for pediatrics in South Africa during the study period^5,9^. The combination of ARV drugs within ART regimens varied; therefore, GRT records with complete regimen data (*n* = 851) were categorized further based on the NRTI contained, i.e., ABC, zidovudine (AZT) and TDF. Regimens included XTC (emtricitabine/lamivudine) and either efavirenz (EFV), PI/r, INSTI or PI/r plus INSTI. For GRT records with complete details of all current ARV drugs in the regimen, we assigned GSSs for individual drugs, i.e., a GSS of 1 for no resistance or potential low-level resistance, 0.5 for low-level resistance or intermediate resistance, and 0 for high-level resistance. The total GSS for each record was calculated by adding the individual drug scores, and a total score ≥2 indicated an active regimen, while a total score <2 suggested reduced potency due to HIVDR^24^. For example, if the patient is taking ABC + 3TC + DTG and a GRT indicates the presence of K65R and M184V mutations, these confer high-level resistance (GSS = 0) to ABC and 3TC, respectively. Therefore, the total GSS for ABC + 3TC + DTG (0 + 0 + 1) is 1, i.e., <2, indicating reduced potency of the regimen for that patient. We did not calculate GSS for GRT records with missing ARV drug regimen data.

### HIVDR geospatial analysis

For each GRT record, we assigned the global positioning system coordinates of the respective healthcare facility at which the GRT specimen was collected, and used the coordinates to create geospatial maps in QGIS 3.30 software^25^. We produced inverse distance weighted (IDW) interpolation maps to illustrate the ARV drug susceptibility levels per facility and to identify spatiotemporal variations in HIVDR levels across KZN. The IDW interpolation approach assumes that unsampled areas near a facility have drug susceptibility values similar to those observed at the facility’s location, as described previously^21^. Sensitivity analyses, conducted by adjusting the power exponent, demonstrated an ideal power of 2 for ARV drug susceptibility IDW map creation.

### Statistical analysis

We used descriptive statistics to assess patterns of HIVDR, GSSs and mutations, and presented the results as percentages in graphs and tables. To evaluate the associations between HIVDR and patient GRT variables, we analyzed continuous variables (i.e., VL and age), estimating odds ratios with cluster-robust standard errors. Standard errors were clustered at the healthcare facility level. Categorical variables (i.e., year of collection, sex and patient status) were analyzed using chi-square tests. GRT records with missing VL data were excluded from the logistic regression analysis.

### Statistics and reproducibility

We conducted all statistical analyses in Stata 18.0 software SE (StataCorp, 2023, College Station, TX, USA) using Stata do-files to ensure reproducibility of results. NHLS CDW provided standardized data from routinely processed GRTs. Uniformly linked facility GIS information from 114 public-sector healthcare facilities served as unique geographic locality “replicates” which could be repeatedly geospatially analyzed by year and district to determine the ARV drug susceptibility levels among CALWH in KZN. For GRTs exhibiting HIVDR, mutations were further categorized based on ARV drug class for cumulative analyses.

### Ethical considerations

We obtained ethics approval from the Biomedical Research Ethics Committee of the University of KwaZulu-Natal (reference number: BREC/00003120/2021). We received further permission from the NHLS Academic Affairs and Research Management System for the use of routinely processed retrospective de-identified HIVDR records for research purposes. As this study used retrospective anonymized HIVDR data, the research ethics committee that approved the study waived the requirement for participant informed consent.

## Results

**Fig. 1:**
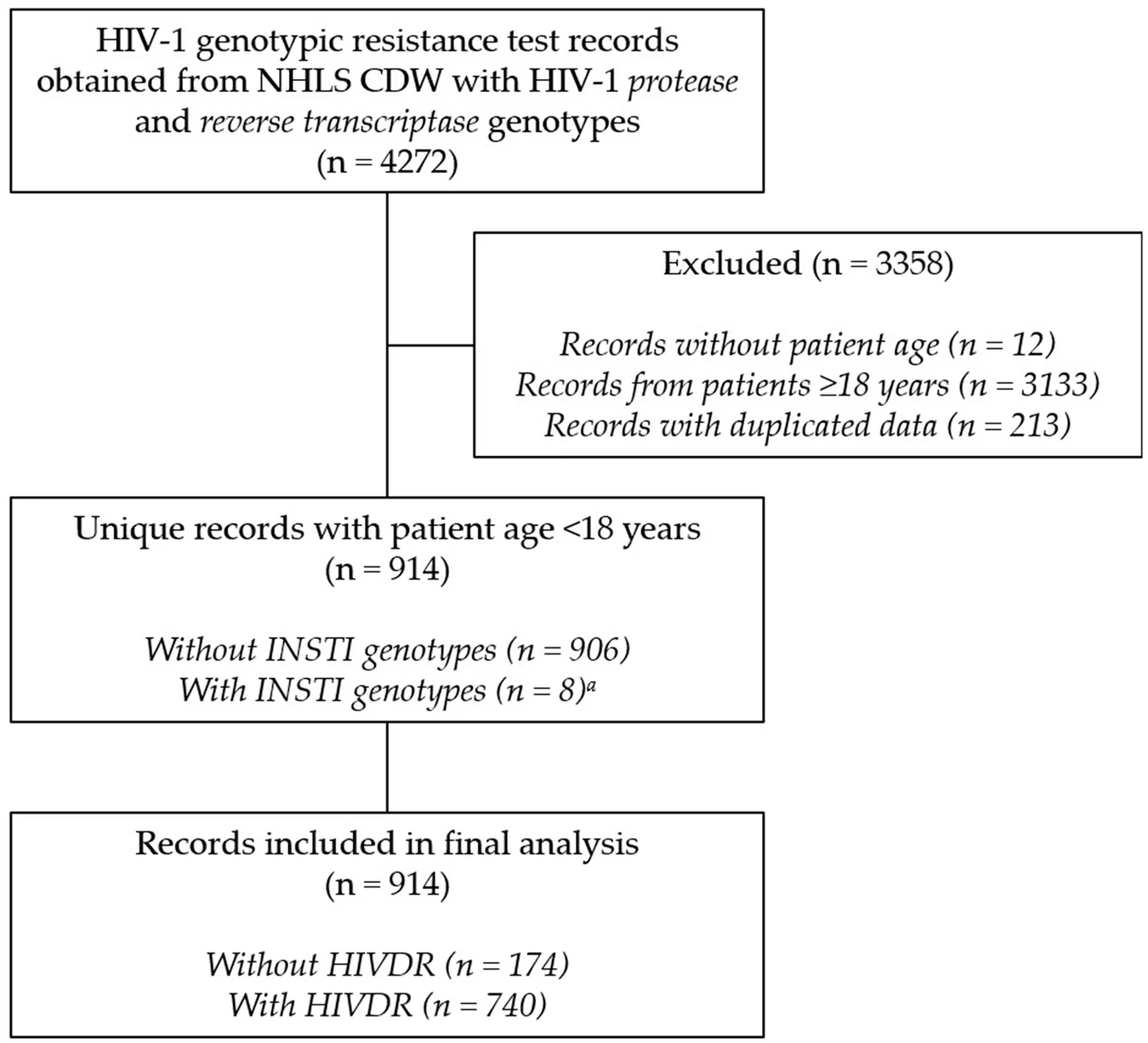
Flowchart of HIV-1 genotypic resistance test records included in final analysis for children and adolescents receiving HIV-1 care in KwaZulu-Natal, South Africa. ^a^Excluded from geospatial analysis due to a small number of INSTI genotypes (*n* = 8). CDW, central data warehouse; HIVDR, HIV-1 drug resistance; INSTI, integrase strand transfer inhibitor; NHLS, National Health Laboratory Service.

Of 914 GRT records obtained from 114 public-sector facilities, all individuals had HIV-1 subtype C infection, with 80.96% (740/914) exhibiting HIVDR. Patients had a median age of 13 years (interquartile range (IQR): 8–16), and 49.34% (451/914) were female. Figure 1 presents a summary of the GRT records that were retrieved and analyzed.

There was no significant difference in the proportion of HIVDR by sex. HIVDR was significantly higher in children (0–9 years) compared to adolescents (10–17 years): 92.22 versus 76.56%, respectively, *p* < 0.05. When compared to adolescents aged 15–17 years, the odds of HIVDR was nearly three times higher in children aged 0–4 years (odds ratio (OR) = 2.94, 95% CI: 1.54–5.60, *p* = 0.001), and over four times higher in children aged 5–9 years (OR = 4.15, 95% CI: 1.94–8.89, *p* < 0.001) (Supplementary Table S1). Seven hundred and eighty GRTs were from children >4 years, of whom 66.2% (516/780) had NNRTI-resistance. One hundred and thirty-four GRTs were from children ≤4 years of age, and of those, 69.4% (93/134) had NNRTI-resistance, 82 of whom had no present exposure to NNRTIs. There were no significant changes observed in HIVDR across the study years (Table 1).

**Table 1:** Characteristics of HIV-1 genotypic resistance tests from children and adolescents living with HIV in KwaZulu-Natal, South Africa

Variable	All GRTs *N* = 914 (%)	*N* = 174 (%)	HIVDR *N* = 740 (%)	*p* value
**Year of collection**				
2018	205 (22.43%)	40 (19.51%)	165 (80.49%)	(0.96)
2019	201 (21.99%)	37 (18.41%)	164 (81.59%)	
2020	223 (24.40%)	45 (20.18%)	178 (79.82%)	
2021	178 (19.47%)	31 (17.42%)	147 (82.58%)	
2022^a^	107 (11.71%)	21 (19.63%)	86 (80.37%)	
**Sex**				
Female	451 (49.34%)	91 (20.18%)	360 (79.82%)	(0.27)
Male	445 (48.69%)	82 (18.43%)	363 (81.57%)	
Not documented	18 (1.97%)	1 (5.56%)	17 (94.44%)	
**Age** in years				
15–17	339 (37.09%)	76 (22.42%)	263 (77.58%)	
10–14	318 (34.79%)	78 (24.53%)	240 (75.47%)	(0.524)
5–9	123 (13.46%)	8 (6.50%)	115 (93.50%)	**(**<**0.001)**
<5	134 (14.66%)	12 (8.96%)	122 (91.04%)	**(0.001)**
**VL** in log_10_ copies/mL, median (IQR)^b^	4.80 (4.26–5.31)	4.91 (4.59–5.20)	4.71 (4.18–5.33)	(0.058)
**Patient status**				
Inpatients/admissions	97 (10.61%)	17 (17.53%)	80 (82.47%)	(0.16)
Outpatients	817 (89.39%)	157 (19.22%)	660 (80.78%)	

*GRT* genotypic resistance test, *HIVDR* HIV-1 drug resistance, *IQR* interquartile range, *mL* milliliter, *VL* viral load.

*P* values for year of collection, sex and patient status were generated using Chi-square tests, while *p* values for age group and viral load were generated using logistic regression with cluster-robust standard errors. Bold *p* values indicate statistically significant results.

^a^Year of collection refers to the year in which each genotypic resistance test (one record per patient) was requested. For the year 2022, the data represent the first half of the year, as the study period concluded on 30 June 2022.

^b^The most recent viral load was not documented for 36 records.

### Patterns and trends of HIV-1 drug resistance

**Fig. 2:**
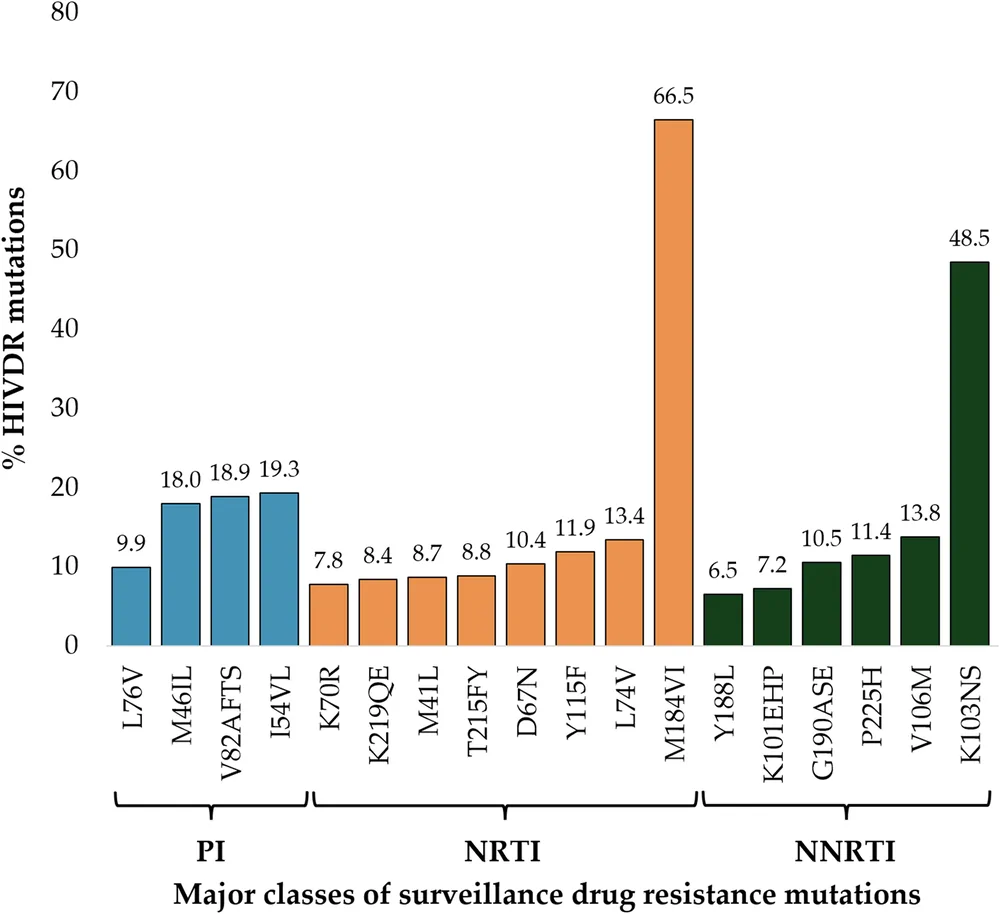
Analysis of specific mutations by drug class identified in 740 HIV-1 drug-resistant genotypes from children and adolescents living with HIV in KwaZulu-Natal, South Africa. The horizontal axis displays major classes of surveillance drug resistance mutations identified in over 6% of HIV-1 drug-resistant genotypes, as classified by the Stanford HIV Drug Resistance Database or the 2022 IAS–USA list of drug resistance mutations^23,26^. HIVDR HIV-1 drug resistance, NNRTI non-nucleoside reverse transcriptase inhibitor, NRTI nucleoside/nucleotide reverse transcriptase inhibitor, PI protease inhibitor.

Among the 740 individuals with confirmed HIVDR, 609 (82.30%) had NNRTI mutations, 525 (70.95%) had NRTI mutations, and 181 (24.46%) had major PI mutations. By ARV drug class, the most frequently detected mutations were the NRTI mutation M184VI (66.49%), the NNRTI mutation K103NS (48.51%), and the PI mutation I54VL (19.32%). The mutation L74V, which confers intermediate resistance to ABC, was detected in 13.38% (99/740) of GRTs, as shown in Fig. 2.

The K65R mutation, which confers resistance to TDF, was identified in only 1.35% (10/740) of GRTs with HIVDR. Of the 9 individuals on DTG-based ART, only 8 GRTs included HIV-1 IN genotyping (1 on DTG <2 years), 7 of which had HIVDR and of those, 3 (37.50%) had resistance mutations to INSTIs. Mutations identified in GRTs exhibiting HIVDR were classified according to drug class using the Stanford HIV Drug Resistance Database and 2022 IAS–USA drug resistance mutations list^23,26^ and are presented in Supplementary Table S2.

Of the 740 with HIVDR, 293 (39.59%) GRTs had single-class resistance (mainly NNRTI resistance), whilst 317 (42.84%) had dual-class resistance, 129 (17.43%) had triple-class resistance, with only 1 (0.14%) having four-drug class resistance. When stratified according to age, ~3 out of every 4 children aged 5–9 years had HIVDR to at least one ARV drug class (86/155; 74.78%), while the proportions among those aged 0–4, 10–14 and 15–17 years were 72.13% (88/122), 59.16% (142/240) and 49.81% (131/263), respectively. The overall proportion of triple-class resistance reduced from 25.45 in 2018 to 15.12% in 2022. Temporal changes in drug class resistance among CALWH are shown in Supplementary Fig. S1.

### HIV-1 genotypic susceptibility

The majority of GRTs were from individuals on PI/r-based ART (802/914; 87.75%), comprising LPV/r or ritonavir-boosted atazanavir (ATV/r). When comparing regimens containing AZT (*n* = 434) and TDF (*n* = 26) to ABC-based regimens (*n* = 391), there was a 23.48% reduction in the odds of HIVDR in patients on TDF-based regimens (OR = 0.77, 95% CI: 0.30–1.97, *p* > 0.58). However, the evidence does not strongly support a protective effect of TDF against HIVDR (Supplementary Table S1). GSSs were predicted only in GRTs with complete regimen data (i.e., 851 out of 914) regardless of whether HIVDR was present. Patients on a regimen containing ABC with EFV or PI/r, and those on an AZT regimen with EFV, had the lowest predicted GSSs (Table 2).

**Table 2:** HIVDR proportions and genotypic susceptibility scores for commonly used antiretroviral regimens in children and adolescents receiving HIV-1 care at public health facilities in KwaZulu-Natal, South Africa, between 2018 and 2022

	All GRTs		HIVDR	Genotypic susceptibility score (%)		
ART regimen^a^	*N* = 851 (%)	*N* = 165 (%)	*N* = 686 (%)	<2	2	>2
**ABC-based**						
ABC + PI/r	339 (39.84)	59 (17.40)	280 (82.60)	203 (59.88)	1 (0.29)	135 (39.82)
ABC + EFV	28 (3.29)	5 (17.86)	23 (82.14)	21 (75.00)	1 (3.57)	6 (21.43)
**AZT-based**						
AZT + PI/r	419 (49.24)	85 (20.29)	334 (79.71)	87 (20.76)	119 (28.4)	213 (50.84)
AZT + EFV	8 (0.94)	0 (0.00)	8 (100.00)	7 (87.5)	1 (12.5)	0 (0.00)
**TDF-based**						
TDF + PI/r	19 (2.23)	4 (21.05)	15 (78.95)	8 (42.11)	0 (0.00)	11 (57.89)

*ABC* abacavir, *ART* antiretroviral therapy, *AZT* zidovudine, *EFV* efavirenz, *GRT* genotypic resistance test, *HIVDR* HIV-1 drug resistance, *PI/r* boosted protease inhibitor, *TDF* tenofovir disoproxil fumarate.

^a^All regimens include XTC, which is either emtricitabine or lamivudine. PI/r refers to ritonavir-boosted atazanavir or lopinavir, and the PI with the lowest identified penalty score was used^23^. GRTs were done at the time of antiretroviral treatment failure. The regimens listed in this table were those administered to at least 1% of the children and adolescents living with HIV with complete ART regimen data.

The highest GSSs were predicted among those on ABC with AZT and a PI/r; however, this regimen is not used as standard of care in South African treatment guidelines (Supplementary Table S3). Only 8 GRTs were from CALWH on INSTIs, 6 of which were on DTG-based ART and of those, 66.67% (4/6) had GSSs >2. Details of HIVDR proportions and GSSs for all other regimens are shown in Supplementary Table S3.

### HIVDR geospatial analysis

**Fig. 3:**
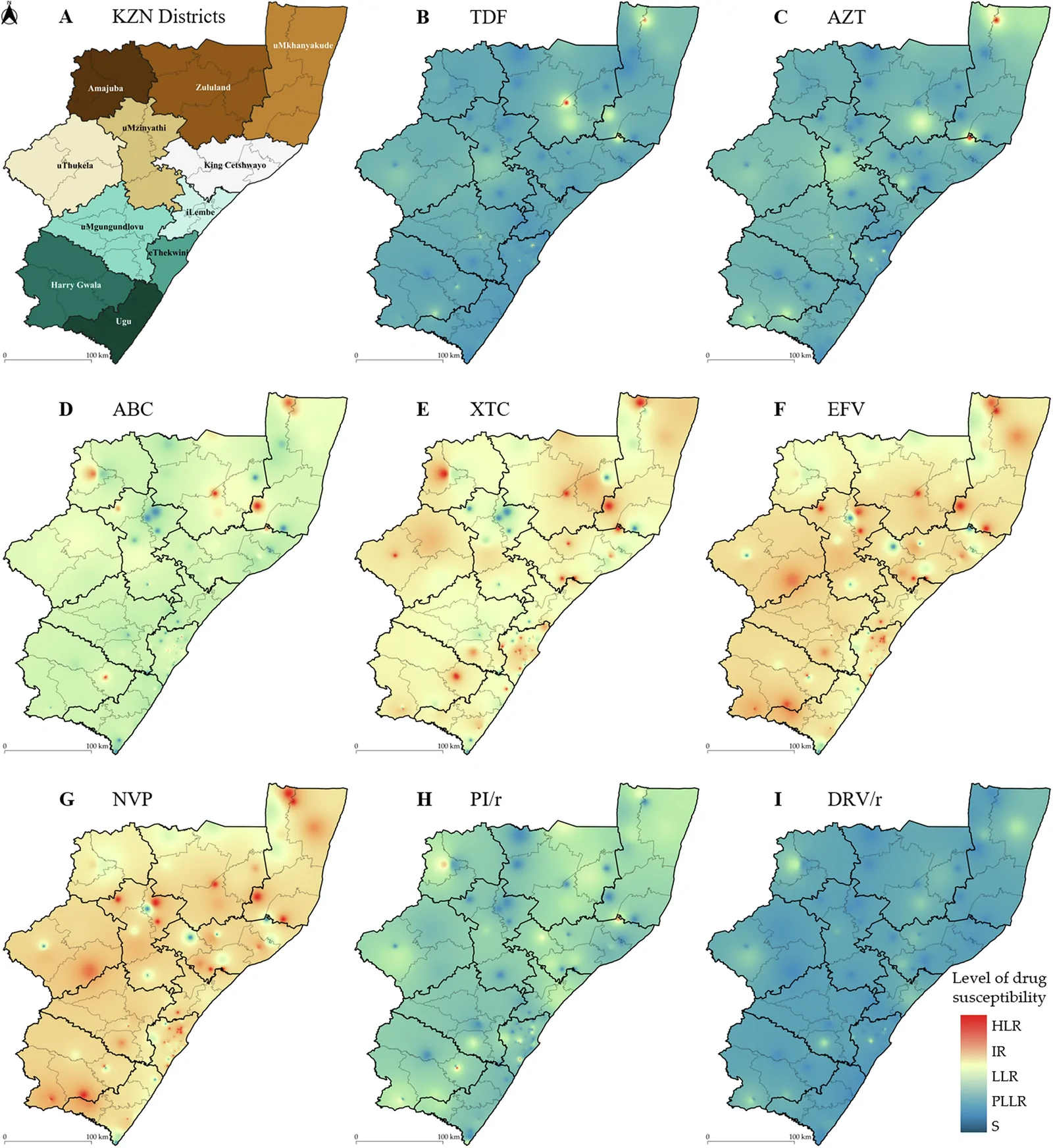
Levels of antiretroviral drug susceptibility among children and adolescents with virological failure in KwaZulu-Natal (KZN) province, South Africa. **A** Map of KwaZulu-Natal showing district names. Interpolation maps showing antiretroviral drug susceptibilities to: **B** TDF tenofovir disoproxil fumarate; **C** AZT zidovudine; **D** ABC abacavir; **E** XTC emtricitabine/lamivudine; **F** EFV efavirenz; **G** NVP nevirapine, **H** PI/r ritonavir-boosted protease inhibitor lopinavir/atazanavir; **I** DRV/r ritonavir-boosted darunavir. Red-blue spectrum represents level of drug susceptibility: HLR high-level resistance, IR intermediate resistance, LLR low-level resistance, PLLR potential low-level resistance, S susceptible. Black lines indicate boundaries of districts and subdistricts. Basemap reissued under CC BY license with permission from Carto Builder user Lilishia Gounder, original ©2025. Accessible from: https://pinea.app.carto.com/map/4745a7dc-a951-4edc-b6b0-169e59f9d718.

GIS mapping using IDW interpolation revealed general uniformity in drug susceptibilities across the 11 districts of KZN province (Fig. 3). Overall, the majority of districts ranged from susceptible to potential low-level resistance to TDF, AZT and ritonavir-boosted darunavir (DRV/r), with low-level resistance observed to boosted PI drugs (namely LPV/r and ATV/r). Furthermore, most districts showed low-level to intermediate resistance to ABC; however, five out of the six localized areas of high-level resistance were observed in northern KZN. Notably, there was intermediate to high-level resistance to the commonly used XTC and the NNRTI drugs, EFV and nevirapine (NVP), despite only 4.16% (38/914) of CALWH having prior exposure to NNRTI-based regimens. Maps with ARV drug susceptibility levels for TDF, AZT, ABC, XTC, EFV, NVP, PI/r (i.e., LPV/r or ATV/r) and DRV/r are shown in Fig. 3.

## Discussion

This retrospective study of 914 routine HIV GRTs from children and adolescents in KwaZulu-Natal, South Africa, showed that HIVDR was three to four times higher in GRT records from children aged 0–9 years when compared to adolescents aged 15–17 years. This is consistent with results from routine GRTs processed between November 2017 and December 2018, which indicated a higher prevalence of PI resistance mutations in children compared to adolescents (43% versus 26%)^27^ in KZN. Geospatial analysis of all GRTs revealed that most districts across KZN showed low-level resistance to PI/r drugs despite 9 out of 10 CALWH receiving PI/r-based regimens, consistent with the well-established high genetic barrier to resistance associated with PIs. Overall, the temporal trends of dual- and triple-class resistance were identified in 3 out of every 5 children. These trends are concerning, given the limited number of ARV drug classes available for optimization of second- and third-line ART in South Africa. Notwithstanding the shift to more potent TDF- and DTG-based regimens from 2018 to 2022, the prevalence of resistance remained unchanged. Only 8 GRTs in this study were from CALWH on DTG-based ART; however, 62.50% showed GSSs ≥2, indicating sustained efficacy of the regimen.

HIVDR was predominantly driven by NNRTI mutations, with almost 80% of GRTs exhibiting NNRTI resistance, despite <5% of individuals being on an NNRTI-based treatment regimen. Four out of every five patients on EFV-containing regimens had total GSSs <2, indicating reduced regimen potency, largely due to NNRTIs having a low genetic barrier to resistance^28^. A systematic review and meta-analysis describing global HIVDR in CALWH over the past decade, noted pooled NNRTI resistance prevalences of 28.38% [18.74–39.08] and 65.17% [53.95–75.63] among ART-naïve and ART-experienced CALWH, respectively^29^. When we compared children ≤4 years to those >4 years in our study, the prevalence of NNRTI resistance was 69% versus 66%, respectively. Of children ≤4 years, 61% had no present exposure to NNRTI drugs as part of their regimen. Several South African studies have highlighted the increasing prevalence of HIVDR in CALWH, primarily due to maternally transferred NNRTI-associated resistance^3,16,30,31^. These studies emphasize the urgent need for continued monitoring of this phenomenon as maternal ART guidelines transition to a DTG-based regimen^3,16,30,31^.

Across all districts of KZN, there was low-level to intermediate resistance to ABC, commonly used in first-line ART regimens for children aged 0–9 years. Five out of the six notably dispersed localities of high-level resistance to ABC were found in northern KZN districts, suggesting the need for targeted programmatic interventions in this region, which is historically known for HIV-1 hyperendemicity^32–34^. The interpolation maps for TDF, AZT and DRV/r demonstrated susceptible levels to potential low-level resistance homogeneously across KZN, whereas LPV/r and ATV/r maps showed predominantly low-level resistance. These geospatial analyses demonstrated variations in ARV drug susceptibility levels, emphasizing the need for enhanced HIV-1 treatment monitoring in CALWH. Even in seemingly uniform regions, geospatial analysis of HIV GRT data can uncover localized areas of high-level resistance to ARV drugs. Spatial analyses can inform, for example, the provision of on-site training to healthcare workers within high-level resistance localities to reduce referral of patients to specialist healthcare facilities for timely HIV-1 GRT and ART regimen switching. This work in CALWH, along with our previous research on HIVDR among adults in KZN^21^, demonstrates how geospatial analysis may be used to inform data-driven public health decision-making.

The predominant NRTI mutation identified was M184VI, present in two-thirds of all GRTs, followed by L74V, detected in 13.4% of GRTs and causing intermediate resistance to ABC. Only ~1% of GRTs had the K65R mutation, which confers low-to-intermediate level resistance to TDF. However, of 27 GRTs from CALWH on TDF-containing regimens, 37.0% (10/27) had a GSS of <2, indicative of HIVDR with reduced regimen potency. The NNRTI mutation, K103NS, was found in over half of all GRTs, resulting in high-level resistance to EFV and NVP. Similarly, high levels of NNRTI-resistance between 65% and 95% were noted in a study among CALWH under 16 years of age^3^, and the proportion among children under 18 months was 53%^2^. Our data imply transmitted resistance with persistent NNRTI mutations in the absence of drug pressure, as demonstrated in early studies where K103N persisted for years in women who received a single-dose NVP for PVT^35,36^. In these young children, it is challenging to differentiate transmitted resistance from acquired resistance due to poor adherence, and analyzing drug concentrations in hair as an objective measure of adherence may prove useful^37^. The most frequently detected PI-specific mutation was I54V (19.1%), which causes low-level resistance to LPV/r while preserving full susceptibility to DRV/r, demonstrating the viability of second-generation PIs when customizing future treatment options for CALWH with HIVDR.

Despite the rollout of DTG in December 2019, there has been a lag in transitioning CALWH to DTG-containing regimens in South Africa. The National treatment guidelines were revised in 2023 to include dispersible DTG tablets for improved dosing in pediatric patients^11^; however, the availability of these dispersible tablets remains limited. While there is ample evidence that DTG is a potent INSTI with a high genetic barrier to resistance^38,39^, it is essential that the NRTIs combined with DTG are fully active, to prevent functional DTG monotherapy, and preserve its long-term use in HIV-1 treatment programs for CALWH. GRT data provides clear evidence on ARV drug susceptibility levels^23^, contributing to improved ART outcomes among CALWH^40^. Given that two out of every five ART-naïve and four out of every five ART-experienced CALWH in Southern Africa have HIVDR^16^, it is imperative that GRTs are made more accessible to CALWH with viremia.

Similar to our previous geospatial studies^20,21^, our findings should be interpreted with regard to the following limitations. Data regarding previous ARV drug regimens, adherence and ART duration were not consistently documented, and we did not merge serial GRTs, if present, within the HIVDR database. We did not account for potential time-varying confounders in the analysis, particularly changes in patient age. Our geospatial analyses used GIS records of facilities rather than household locations, based on the assumption that CALWH obtained a GRT at the facility closest to their home. We did not consider district/facility HIVDR testing practices, nor contextualize our geographical analysis within the local CALWH population, nor account for ART coverage by district, subdistrict or facility. Given that only 8 CALWH were on DTG-based regimens, we could not geospatially analyze drug susceptibility levels for DTG, nor could we conduct a stratified analysis comparing pre-DTG and post-DTG transition phases. Therefore, similar studies are needed, ideally addressing the limitations identified in this study, to better understand the burden of HIVDR and inform ART decision-making. There is a notable confounder and potential bias in that only select individuals with virological failure had genotypic testing. A genotypic methodological limitation is that the sequencing assay is designed to detect subtype C virus in plasma specimens with VLs ≥1000 copies/mL. Lastly, future research should ideally include integration of maps with PVT and pediatric HIVDR data to find linked HIVDR problems among antenatal and infant populations in specific geographic regions.

## Conclusions

By integrating routine HIV-1 GRT records with GIS analysis, we identified concerningly high levels of HIVDR among younger children. This underscores the urgent need for policies that support near-real-time surveillance of HIVDR, to enable targeted treatment interventions to prevent HIVDR mutations and timely ART switching for CALWH experiencing virological failure. Moreover, the use of dispersible DTG-based regimens for CALWH should be expedited to improve overall ART coverage and achieve better virological suppression by using more palatable and effective ART regimens. Geospatial mapping revealed geographic variations in ARV drug susceptibility levels across KZN, highlighting the potential use of geospatial analysis in HIVDR surveillance to support data-driven public health decision-making.

## Supplementary information

Transparent Peer Review file

Supplementary Information

Description of Additional Supplementary Files

Supplementary Data 1

## Data Availability

Raw data were generated through routine genotypic testing at the Department of Virology, National Health Laboratory Service facility in Durban, South Africa. All relevant data and Stata do-files are available from the authors upon reasonable request. Requests for data should be addressed to L.G. at gounder@ukzn.ac.za. Additionally, the HIVDR database supporting the findings of this study has been made available as a dataset in the supplementary information files.
