# Supplementary material for: Geospatial analysis reveals similar HIV-1 drug resistance patterns among antiretroviral therapy-experienced children and adolescents (0-17 years) of South Africa
Source: Commun Med (Lond). 2026 Jul 7;6:570. doi: 10.1038/s43856-026-01770-9 (PMC13720034; doi:10.1038/s43856-026-01770-9)
Supplement: Supplementary file 1 [file 43856_2026_1770_MOESM1_ESM.pdf]

nature portfolio

Peer Review File

Geospatial analysis reveals similar HIV-1 drug resistance patterns among antiretroviral therapy-experienced children and adolescents (0-17 years) of South Africa

Corresponding Author: Dr Lilishia Gounder

This file contains all reviewer reports in order by version, followed by all author rebuttals in order by version.

Version 0:

Reviewer comments:

Reviewer #1

(Remarks to the Author)
I co-reviewed this manuscript with one of the reviewers who provided the listed reports. This is part of the Communications Medicine initiative to facilitate training in peer review and to provide appropriate recognition for Early Career Researchers who co-review manuscripts.

Reviewer #2

(Remarks to the Author)
Thank you for the opportunity to review this manuscript on geospatial surveillance of HIV-1 drug resistance (HIVDR) among children and adolescents living with HIV (CALWH) in KwaZulu-Natal, South Africa, a hyperendemic region with high pediatric HIVDR prevalence. By combining routine genotypic resistance test data with GIS mapping from 2018–2022, the study reveals high levels of HIVDR among CALWH, with younger children disproportionately affected and widespread resistance to multiple drug classes. This work was an interesting read and is relevant for informing HIV treatment policy and optimizing pediatric ART regimens in high-burden settings.
While the HIVDR component is methodologically sound, it does not appear to add significant new insights beyond what is already established in the literature. I encourage the authors to clarify its added value or consider refocusing this section to better highlight how the integration of geospatial analysis improves HIVDR monitoring. Additionally, the geospatial analyses could be developed further, as they have clear potential to yield more novel and impactful findings. Overall, the manuscript would benefit greatly from major revisions to the structure, inclusion of more methodological detail, clearer presentation of results (including well-structured tables), and better integration of the geospatial component as a novel contribution. These improvements would substantially improve the clarity and overall quality of the manuscript.
Major comments :
Overarching comments include that the methods section lack sufficient detail, which makes it difficult to assess the robustness of the analyses. Key aspects, include the approach modelling, require clarity. For example, details on needed on the number of health facilities included and details on the ART regimens participants were exposed to, the handling of missing data, approach to statistical modelling, and geospatial analysis methods used.
Similarly, the results are not always presented in a clear, systematic manner, and important contextual information (such as participant demographics, ART duration, regimen details, and local treatment guidelines) is missing or insufficiently described. Several findings need to be communicated more clearly using improved tables, more precise terminology, and consistent reporting of statistics (for example, OR are reported in the text but not in the tables).
Finally, the discussion would benefit from tighter organization, avoiding repetition of results, expanding comparisons to literature other settings and countries, and ensuring that key interpretations are fully supported by the data presented.
I encourage the authors to carefully address the detailed points below to strengthen the manuscript's methodological transparency, presentation of results, and overall contribution to the field.
1. Title: I suggest clarifying the age group being referred to and specifying what is meant by 'ART experienced.' It may be clearer to indicate how long the children have been on ART or provide a similar description.
Abstract
2. Lines 29 – 30: It is unclear what the authors mean by "…changes in HIVDR…" are they referring to changing trends in HIVDR across different antiretroviral drug classes? or variations by geographic location of the study participants? or

differences based on the ART regimens participants are receiving?
3. Lines 34 – 35 (Results section of the abstract): typically adolescence refers to the age range between 10 and 19 years, and childhood spans from 0 to 9 years. However, the authors reported an age range of 0 to 17 years… Was there no data available for individuals aged 18 and 19? Please provide justification for the chosen age range.
4. What proportion of participants with NNRTI DRMs were actually on these antiretrovirals given that these mutations can persist for a long time due to their prolonged half-lives in the body?
5. Lines 42 to 43: It would be interesting to report the resistance levels to TDF as well, as this NRTI is the backbone of the currently recommended TLD (Tenofovir + Lamivudine + Dolutegravir) regimen.
Introduction
6. Line 72: It is unclear why CALWH aged <3 years are specifically referred to as a composite group. Why was this particular age group singled out, and how is this distinction relevant for the later analyses? Are these children not a distinct age category, separate from adolescents, rather than part of a combined population?
7. Lines 75 and 97: Providing the exact number of CALWH, rather than simply stating "…largest HIV-1 epidemic…" or "…Given the high prevalence of HIVDR among pediatrics in South Africa…", respectively, would help readers better understand the magnitude and relevance of this population.
8. Lines 98 to 99: The authors state that "…there is a critical need for effective near-real time geospatial surveillance of HIVDR in CALWH…," but this claim lacks adequate justification in the introduction section. This comes out of the blue in this section without prior clarification about what they mean by "effective near-real time geospatial surveillance", nor explanation about how this approach would integrate with or enhance existing HIVDR surveillance strategies. This statement would benefit from further clarification and contextual grounding, especially given that it addresses a core aspect explored in the manuscript.
Materials and Methods
9. Paragraph 1: Could the authors specify how many public health facilities were included in the study?
10. Lines 115 to 117: Given that the authors report relatively high pretreatment drug resistance in the study population (lines 89 to 93), could they clarify the justification for waiting two years post-DTG initiation before GRT? A brief justification (if any) on how this recommendation aligns with current WHO or regional guidelines on HIVDR and balances risks and benefits in this specific context would strengthen the manuscript.
11. Line 127: As already mentioned in an earlier comment in the abstract section, it is still unclear why the authors defined adolescence as ages 10 to 17 years instead of the more commonly used age range of 10 to 19 years, as indicated in the reference they provided. Please justify this choice.
12. Figure 1 presents key findings and is already part of the results; therefore, it should be moved to the results section and used to form the first paragraph of this section to ensure proper flow of information.
13. Please include a brief description of the different ART regimens to which the participants included in the analysis were exposed. This information would improve understanding of the interpretation of GRT drug resistance mutations and enhance the public health relevance of the findings, especially in the context of limited availability of ARV drug formulations. This would also improve the understanding of the results provided in lines 193 to 196; lines 208 to 213.
14. Lines 168 to 174 (Statistical analysis): Please provide more information on the variable types including those used in the modelling; were continuous variables like viral loads modeled as continuous or categorized? Did you perform any adjustments or focused just on univariate analysis? The choice of a one-sided chi-square test is statistically unconventional, what was the rationale for this choice? How was missing data handled in your analysis?
15. Statistical analysis:
- There is no need to present both proportions and percentages, presenting percentages alone would be clearer and more consistent.
- Additionally, the section on logistic regression modelling would benefit from more detail. For example, did the authors account for potential clustering by health facility or district? Were possible confounding factors considered when exploring factors associated with drug resistance?
- Finally, did the models adjust for time to account for changes in treatment regimens, drug availability, and other relevant programmatic shifts?
- Could the authors provide additional details regarding the geospatial analysis to enhance the clarity and reproducibility of the study?
Results
16. The authors should include a clear description, early in the manuscript, of the characteristics of the CALWH for whom the 914 records were available, for example, their age distribution, sex, and other relevant demographics. This is essential to properly contextualize the subsequent risk factor analyses.
17. Lines 188 – 189:
- The sentence 'Notably, the proportion of HIVDR was significantly higher in children compared to adolescents (92.22% versus 76.56% respectively)…' presents percentages, as such, I suggest replacing 'proportion' with 'percentage' for accuracy.
- I also suggest removing 'Notably' as this implies interpretation of the results, whereas simply stating the facts directly would be clearer in this section.
- Additionally, please add the exact age ranges for 'children' and 'adolescents' to enhance clarity.
18. Lines 189 – 193: To enhance clarity and facilitate interpretation, I recommend including these results in a dedicated table. I suggest a Table 2 presenting the odds ratios along with clear information about the variables adjusted for.
19. Table 1:
- If available, please include details on ART treatment duration for the study participants. This information is critical for enhancing the understanding and interpretation of HIVDR, bearing in mind that HIVDR is closely linked to cumulative drug pressure overtime, which drives the selection of drug resistance mutations.
- The label 'Year of collection' needs further clarification. Was there one record per individual for each time period, or multiple records over time?

- Additionally, it would be helpful if the authors could explain how they accounted for potential time-varying confounders, such as changes in age, in their analysis.
- Could the authors please clarify the rationale for the chosen age categorization in this table, especially since the authors report on the <3 years age group?
- Could the authors clarify the meaning of the label ‘Inpatients’ in table 1 to ensure readers fully understand the category?
20. Please note that an odds ratio of 3 does not directly mean that the outcome is ‘three times higher’ in the same way a prevalence ratio does. This misinterpretation must be corrected to ensure accurate reporting of the results. Additionally, all odds ratios should be clearly presented in the tables, not just selectively reported in the text.
21. Lines 231 to 233: The results on multiclass drug resistance would be clearer if the authors included a brief overview of the treatment guidelines recommended, as well as the treatment regimens available in the South African context.
22. Lines 242 – 246: “When comparing regimens containing…” Could the authors clarify or provide more details in the Methods section regarding the analysis mentioned here, as it is not currently described?
23. It would be helpful to provide more detail on the other risk factors considered in the analysis. I suggest including a clear section describing the logistic regression methods used, along with the full output of this modelling, to strengthen the interpretation of your results.
24. Current treatment guidelines recommend switching to DTG-based regimens for pediatric patients. Given this, it would be helpful if the authors could comment on why only 8 participants in the study were on DTG, and discuss any potential implications this may have for the findings..
Discussion
25. Line 301: “…persistent viraemia…” this is challenging to ascertain because the authors did not report treatment duration or the proportions of participants on different antiretroviral drugs. Both of these are important to consider, given the varying genetic barriers of these drugs, and are necessary to fully interpret the resistance patterns observed, as some mutations arise more easily under specific drug pressure.
26. Paragraph 1, lines 301 – 306: I would suggest to focus on providing a summary of the main findings here and to reserve interpretation and discussion of these findings for later in the section.
27. For lines 310–311: please clarify if the comparison between children under 3 years and those over 3 years was explicitly outlined in the methods section to ensure consistency.
28. It would strengthen the discussion to more thoroughly relate the findings to existing literature from other countries, providing a broader comparative context.
29. Consider including an analysis and discussion of results by sex, if possible, as this could provide valuable insights.
Minor comments :
Abstract
30. The abstract could benefit from additional details to enhance clarity for the reader.
- For example, it would be helpful to specify the time period over which trends were assessed, the number of facilities included, the type of genotypic resistance testing (GRT) performed, and the kind of GIS analysis conducted.
- Additionally, in the results section of the abstract, please consider indicating the age range or median age of the 914 CALHW included.
- Finally, since an odds ratio is presented in the abstract, it would be important to clarify in the methods whether a logistic regression was conducted and, if so, to briefly describe it.
31. The abstract mentions GIS mapping in the results section, but it does not specify which locations in KwaZulu-Natal (KZN) had higher or lower levels. Adding more details here would strengthen the conclusion, which currently emphasizes the potential of geospatial analysis for data-driven public health decision-making but lacks specific geographic context.
Plain language summary
32. Line 56: When the authors mention “… younger children…”, which specific age group are they referring to? Please specify this age group in brackets for clarity.
33. Line 58: Specify the age group in brackets here as well for clarity.
Materials and Methods: Study population and design
34. Line 107: Please specify the study design… for example, is it a cohort study?
35. Line 110: Did the authors conduct a stratified analysis comparing the pre-transition and transition phases to DTG, and the effect this transition had on the prevalence of HIVDR?
Results
36. Table 1: With median viral loads this high, did the study assess adherence to treatment in these participants? Major mutations being mostly quasi-species mutations may further indicate adherence challenges (lines 210 to 212).
37. For clarity and consistency, I suggest reporting percentages to one decimal place and presenting p-values in brackets.
38. Table 1: It may be helpful to remove the column header ‘No HIVDR’ as this is already implied by the ‘HIVDR’ column. Additionally, please clarify which statistical test was used to generate the p-values shown, as this information would help readers interpret the results more easily.
Discussion
39. Lines 298 to 301: “… Comparable results… in KZN.” Please rephrase this sentence for clarity, as the purpose and meaning of the comparison are not clearly conveyed.
40. Line 308: Please adjust this to “HIVDR was predominantly driven by NNRTI mutations,…” Additionally, do the South African guidelines still recommend NNRTIs or have these been phased out in the current recommendations?
41. Line 310: “When comparing children less than 3 years…”
42. Line 328: the phrase “These geospatial analyses maps…” could be simplified by removing the word “maps” for clarity.
43. There is repetition of results in the discussion section; it would be beneficial to focus more on comparing and contrasting these findings with those from other studies to highlight their significance.

General comment
44. There are too many acronyms used throughout the manuscript, including in the abstract. I suggest considering reducing

the number of acronyms where possible to enhance readability and make the text more accessible for a broader audience.

Reviewer #3

(Remarks to the Author)
The statistical analysis is well explained and appropriate for the study.

Reviewer #4

(Remarks to the Author)
Summary
In the article entitled “HIV-1 drug resistance among ART-experienced children and adolescents in KwaZulu-Natal, South Africa: Geospatial analysis” Gounder et al have assessed the prevalence and geospatial trends of HIV drug resistance (HIVDR) mutations amongst the children and adolescents living with HIV (CALWH) in the KwaZulu-Natal region of South Africa between 2018-2022. The authors have found the HIVDR to be more prevalent in children (0-9 years) compared to adolescents (15-17 years) with sustained virological burden. Higher levels of HIVDR to lamivudine, nevirapine and efavirenz were observed based on the geospatial analysis across KZN province. The article has highlighted the importance of real time geospatial trend analysis for effective targeted programmatic interventions in the hyperendemic regions. This has relevance not only for south Africa with highest burden of HIV but also globally, with the HIV pandemic now being driven through specific transmission clusters amongst high-risk populations.

Overall impression of the work
The article is well written, largely descriptive in nature and includes comprehensive statistical analyses of HIVDR, HIVDR distribution trends across geographical locations, age groups and genders. The data provided is however limited by the availability of comprehensive case information in the resource limited setting of KZN. The authors have included and addressed most of the confounders in the discussion section adequately. As discussed by the authors, the inconsistencies in refering cases for GRT in spite of high viral load (>1000 copies/mL) is a major limitation of this work that limits its impact.

Specific comments, with recommendations for addressing each comment
1. It is unclear what the overall burden of CALWH at each of the 11 districts in the KZN region is. Based on the supplementary data provided the range of GRT data varies between 15 (Harry Gwala) to 324 (eThekwini), cumulatively over the study period of 5 years. This would be important to delineate whether the included cases are proportionately representative of the geospatial trends. If not, this needs to be included and discussed as a potential limitation of the study with inclusion of district-wise distribution of cases.
2. The authors should clarify if any longitudinal sampling was considered from any of the study participants.
3. The authors have described the participant grouping to be as follows (line 151), (i) 0-3 years, (ii) 4-9 years, (iii) 10-14 years and (iv) 15-17 years. The authors have also consistently differentiated the observations related to the 0-3 age group from the others. However, Table 1 with statistical analyses refers to different age groups. Uniform grouping would make the trends clearer.
4. The article would benefit from further elaboration on the genotypic susceptibility scores section (lines 154-158) preferably with an example. Are the scores included for only the drugs taken by the participants or all of the drug classes as predicted by the Stanford HIV database? This isn’t clear in the supplementary files.
5. Utility of geospatial trend analysis for targeted interventions is logical. The authors have discussed dispersed sections of northern KZN districts having higher abacavir resistance prevalence (lines 325-327). However, the presented case does not seem convincingly in support of the geospatial analysis. The authors may discuss the potential explanation for this observation and how it can be addressed to make it more impactful.

Reviewer #5

(Remarks to the Author)
REVIEW REPORT
Review_summary
Review summary
English language
( ) I am not qualified to assess the quality of English in this paper
( ) English very difficult to understand/incomprehensible
( ) Extensive editing of English language required
( ) Moderate editing of English language required
(X) Minor editing of English language required
( ) English language fine. No issues detected

COMMENTS
The draft manuscript by Gounder et al, entitled “HIV-1 drug resistance among ART-experienced children and adolescents in KwaZulu-Natal, South Africa: Geospatial analysis” addresses a critical issue of HIVDR among children and adolescents in South Africa. The analysis has been done with a large dataset of 914 GRTs among CALWH in a hyperendemic setting. The integration of geospatial analysis is particularly innovative and an inspiration for health researchers in the region. The study highlights a concerningly higher prevalence of HIVDR among CALWH across KZN. These useful findings are crucial in informing policy not just within South Africa but in similar settings. The study also highlights interesting policy gaps in the

monitoring of HIVDR among CALWH.
SUGGESTIONS FOR IMPROVEMENT
I would like to highlight the following observations to the authors.
1. While the title is clear and concise, I I suggest amending it to, "….: A geospatial analysis" to improve readability
2. In the abstract, I suggest highlighting the sample size in the methods section. E.g. We analyzed 914…
3. In line 45, "general uniformity" could be modified to read as "overall uniformity with variations in regions"
4. The statement in lines 66-70 could be shortened to improve readability.
5. The authors highlight that South Africa is home to the largest pediatric HIV epidemic globally. This could be backed by current statistics and reference (s).
6. In the methods section, I commend the authors for clearly describing the study design. However, I would suggest inclusion of subheadings such as study population, laboratory methods, geospatial mapping, to improve readability.
7. GSS is a technical concept that most non-specialist readers may find difficult to understand. I suggest authors describe what it is in a simplified language in the methods section.
8. The legends seem to be in the same font size as the rest of the text, and it may be difficult for readers to distinguish. I suggest modifying the texts of legends to distinguish them from the main text of the draft manuscript.
9. While the results appear with well-structured tables and figures, I suggest the following improvements:
i) In figure 2, the word major classes of surveillance drug resistance mutations (SDRMs) should be included in the horizontal axis.
ii) The line(s) at the horizontal axis of figure 2, indicating where PI, NNRTI, and NRTI end and or begin looks like a continuous line. I suggest it is redesigned to show distinction between the SDRMs.
iii) Authors should revise the results section and move statements of interpretation of results to the discussion section, instead. E.g. line 263… "Lower GSSs in patients on EFV containing regimens are largely due to NNRTIs low genetic barrier to resistance"
10. In the discussion section, the authors thoroughly interpreted the results and included relevant prior studies. The authors highlight clinical implications such as the need for real-time HIVDR surveillance. However, I suggest the following for improvement:
i) A clear structure be followed in the discussion to encompass main findings, comparative studies, explanations and implications.
ii) The narrative be streamlined to avoid the discussion appearing like a blend of results and literature review. Focus should be given to policy and clinical implications.
iii) Some sentences are too lengthy and could be revised to improve readability.
11. There is a need to proof-read the whole manuscript to eliminate typos e.g. line 349…due [to] poor adherence…
12. In the conclusion section, line 389, the authors used the word 'significantly' without quoting the statistical p value. I suggest that this be revised. E.g. "By integrating routine HIV-1 GRT records with GIS analysis, we identified significantly (remarkably/ concerningly) higher levels of HIVDR among younger children.
Recommendation
This draft manuscript is interesting and, in my opinion, worthy of publication. It would, however, require moderate revision to address the issues I have suggested above.

Jonah Omooja,
Uganda Virus Research Institute, Entebbe,
Department of General Virology,
cjomooja@uvri.go.ug

Makerere University, Kampala
Department of Molecular Biology and Immunology,

Version 1:

Reviewer comments:

Reviewer #1

(Remarks to the Author)
I co-reviewed this manuscript with one of the reviewers who provided the listed reports. This is part of the Communications Medicine initiative to facilitate training in peer review and to provide appropriate recognition for Early Career Researchers who co-review manuscripts.

Reviewer #2

(Remarks to the Author)
Thank you for the opportunity to review the revised version of this manuscript. I appreciate the authors' efforts in addressing many of the comments raised during the first round of review. The revisions have improved several aspects of the manuscript, particularly the clarification of the study population, the use of terminology related to HIV drug resistance, and the specification of the antiretroviral regimens to which participants were exposed. The authors have also provided additional detail on the national treatment guidelines and made efforts to improve the clarity of the methodology and the

overall presentation of the study. The geospatial component has likewise been strengthened, although some aspects still require further clarification.
The topic remains relevant, as the manuscript explores the use of geospatial approaches to better understand patterns of HIV-1 drug resistance (HIVDR) among children and adolescents living with HIV in a high-burden, resource-limited setting. The analysis of genotypic resistance data is thorough and provides valuable information on resistance patterns and prior treatment exposure in this vulnerable population. However, the added value of the geospatial component is less clear. While mapping resistance patterns is a promising approach in principle, the manuscript does not yet fully demonstrate how this integration meaningfully strengthens surveillance or leads to actionable implications for treatment strategies in this context. Further clarification is needed on how the spatial analysis contributes beyond descriptive mapping and how it could realistically inform programmatic decision-making in similar settings.
While the revisions have improved the manuscript, several points would still benefit from clarification and adjustments to enhance overall clarity and consistency. These are outlined below for the authors' consideration. Overall, I would support acceptance after these revisions. In particular, the authors should clarify the central focus of the manuscript: either emphasize the strong genotypic resistance analysis with the geospatial component as supportive, or further strengthen the geospatial analysis and clearly demonstrate its added value for surveillance and treatment strategies, as currently suggested.
Minor comments :
Title: The abbreviation ART appears in the title but is not defined. Consider spelling it out as antiretroviral therapy (ART) to improve clarity and comply with journal conventions regarding abbreviations.
Line 40 (Results section of the abstract) – report the interquartile range (IQR) of the median age of the study participants.
Lines 40 – 42 (Results section of the abstract) – the sentence "Of the 740 with HIVDR, 609 (82.30%) have non-nucleoside reverse transcriptase inhibitor (NNRTI) mutations with 32/609 (5.25%) on NNRTIs…" is somewhat difficult to follow. Restructure this sentence for improved clarity and readability. In addition, please ensure that the appropriate past tense is used when reporting results.
Line 52 (Conclusions section of the abstract) – the phrase "… demonstrate higher…" would benefit from clarification. Specify higher compared to what reference group or comparison ?
Introduction: the manuscript highlights a "crucial" or "urgent" need for GIS-linked HIVDR monitoring. While the authors attempt to clarify this point, the justification could be strengthened further. The introduction presents a compelling rationale for improving HIVDR surveillance among this vulnerable population; however, it remains somewhat unclear how GIS-based approaches specifically enhance current monitoring strategies. Expanding this section to briefly discuss the current state of the art in GIS applications for HIVDR or infectious disease surveillance, and citing relevant studies where similar approaches have been used, would help better substantiate the proposed value of GIS-linked monitoring in this context. At present, this is not sufficiently addressed in the manuscript and would benefit from further development.
Additional comments (remaining issues from previous review round):
• Abstract (Lines 29-30): In the previous review, I requested clarification on what was meant by "changes in HIVDR." The authors have replaced this with "patterns of," which improves wording but does not fully resolve the underlying ambiguity. It remains unclear whether this refers to variation by drug class, geographic location, or ART regimen. Please specify what "patterns" refers to in this context.
• Title: The inclusion of the age (children and adolescents) in the title was suggested in the previous round of review but has not been addressed. Given that the study population is specific and central to the manuscript, the age of the population included should be reflected in the title.
• Statistical analysis (Lines 193-200): The statistical analysis section remains limited in detail. In particular, the authors should specify what clusters were used (i.e. was it the health facilities) for the cluster-robust standard errors. In addition, the sentence describing the logistic regression analysis (lines 195 - 199) would benefit from rephrasing for clarity. As currently written, it is difficult to follow, particularly regarding how continuous and categorical variables were handled and incorporated into the models. Clarifying the analytical approach would improve transparency and reproducibility.
Overall, I commend the authors for the revisions made in response to the previous review comments. Addressing the points above would further improve the clarity and contextual framing of the manuscript.

Reviewer #4

(Remarks to the Author)
The article entitled "HIV-1 drug resistance among ART-experienced children and adolescents in KwaZulu-Natal, South Africa: Geospatial analysis" reports on trends of higher HIVDR in younger CALWH in KwaZulu-Natal, South Africa. The study suffers from inherent limitations related to unequal sampling, reporting of HIVDR cases as well as lack of some key data. However, through the revised manuscript these have now been adequately indicated and discussed.

Reviewer #5

(Remarks to the Author)
I commend other reviewers for the comments they raised that have consequently improved the manuscript remarkably. I want to thank the reviewers for dedicating time to revise the manuscript.

The authors have satisfactorily addressed the comments I raised earlier.

The discussion has been revised thoroughly, and ideas are well-articulated. However, in the revised manuscript, I noticed that the discussion has subheadings. While I suggested a well-structured flow of ideas in the discussion, I would not recommend subheadings within the discussion. I suggest the authors delete these subheadings to allow a natural flow of the

discussion.

Version 2:

Reviewer comments:

Reviewer #1

(Remarks to the Author)
I co-reviewed this manuscript with one of the reviewers who provided the listed reports. This is part of the Communications Medicine initiative to facilitate training in peer review and to provide appropriate recognition for Early Career Researchers who co-review manuscripts.

Reviewer #2

(Remarks to the Author)
Thank you for letting me review the revised manuscript, "Geospatial analysis reveals similar HIV-1 drug resistance patterns among antiretroviral therapy-experienced children and adolescents (0-17 years) of South Africa." This manuscript looks at HIV-1 drug resistance patterns in children and adolescents on antiretroviral therapy in KwaZulu-Natal, South Africa. It also examines how these resistance profiles are spread across the region. The topic is important because HIV drug resistance remains a challenge for adolescents and young people, and better surveillance is needed in high-burden settings. The authors have addressed the comments raised during the previous rounds of review. They clarified the study population and title, improved how they present and interpret the resistance data, explained the statistical methods more clearly, and added helpful context about the geospatial part of the study. The issues from the last review have been resolved, and the manuscript is now much clearer and easier to follow.

Open Access This Peer Review File is licensed under a Creative Commons Attribution 4.0 International License, which permits use, sharing, adaptation, distribution and reproduction in any medium or format, as long as you give appropriate credit to the original author(s) and the source, provide a link to the Creative Commons license, and indicate if changes were made.
In cases where reviewers are anonymous, credit should be given to 'Anonymous Referee' and the source.
The images or other third party material in this Peer Review File are included in the article's Creative Commons license, unless indicated otherwise in a credit line to the material. If material is not included in the article's Creative Commons license and your intended use is not permitted by statutory regulation or exceeds the permitted use, you will need to obtain permission directly from the copyright holder.
To view a copy of this license, visit https://creativecommons.org/licenses/by/4.0/

RESPONSE TO REVIEWER COMMENTS

Geospatial analysis reveals similar HIV-1 drug resistance patterns among ART-experienced children and adolescents of South Africa

General response: Thank you for the comprehensive and insightful comments for the above-mentioned manuscript. We appreciate and have addressed each comment as shown in the responses below. All changes that have been made to the manuscript are shown as tracked changes and highlighted in yellow with line numbers referring to the revised "tracked" version of the manuscript.

Reviewer 1
I co-reviewed this manuscript with one of the reviewers who provided the listed reports. This is part of the Communications Medicine initiative to facilitate training in peer review and to provide appropriate recognition for Early Career Researchers who co-review manuscripts.

Response to Reviewer 1
Thank you for reviewing the manuscript.

Reviewer 2

General Comments

Thank you for the opportunity to review this manuscript on geospatial surveillance of HIV-1 drug resistance (HIVDR) among children and adolescents living with HIV (CALWH) in KwaZulu-Natal, South Africa, a hyperendemic region with high pediatric HIVDR prevalence. By combining routine genotypic resistance test data with GIS mapping from 2018–2022, the study reveals high levels of HIVDR among CALWH, with younger children disproportionately affected and widespread resistance to multiple drug classes. This work was an interesting read and is relevant for informing HIV treatment policy and optimizing pediatric ART regimens in high-burden settings.

While the HIVDR component is methodologically sound, it does not appear to add significant new insights beyond what is already established in the literature. I encourage the authors to clarify its added value or consider refocusing this section to better highlight how the integration of geospatial analysis improves HIVDR monitoring. Additionally, the geospatial analyses could be developed further, as they have clear potential to yield more novel and impactful findings. Overall, the manuscript would benefit greatly from major revisions to the structure, inclusion of more methodological detail, clearer presentation of results (including well-structured tables), and better integration of the geospatial component as a novel contribution. These improvements would substantially improve the clarity and overall quality of the manuscript.

Major comments :

Overarching comments include that the methods section lack sufficient detail, which makes it difficult to assess the robustness of the analyses. Key aspects, include the approach modelling, require clarity. For example, details on needed on the number of health facilities included and details on the ART regimens participants were exposed to, the handling of missing data, approach to statistical modelling, and geospatial analysis methods used.

Similarly, the results are not always presented in a clear, systematic manner, and important contextual information (such as participant demographics, ART duration, regimen details, and local treatment guidelines) is missing or insufficiently described. Several findings need to be communicated more clearly using improved tables, more precise terminology, and consistent reporting of statistics (for example, OR are reported in the text but not in the tables).

Finally, the discussion would benefit from tighter organization, avoiding repetition of results, expanding comparisons to literature other settings and countries, and ensuring that key interpretations are fully supported by the data presented.

I encourage the authors to carefully address the detailed points below to strengthen the manuscript's methodological transparency, presentation of results, and overall contribution to the field.

Response to Reviewer 2

Thank you for reviewing the manuscript and for the detailed comments. We have made the following changes to the manuscript:

**Specific Comments**	**Response**

1. Title: I suggest clarifying the age group being referred to and specifying what is meant by ‘ART experienced.’ It may be clearer to indicate how long the children have been on ART or provide a similar description.	1. Title: We have adapted the title as recommended by Communications Medicine. Page 1 (line 1-2); “Geospatial analysis reveals similar HIV-1 drug resistance patterns among ART-experienced children and adolescents of South Africa”
2. Abstract: Lines 29 – 30: It is unclear what the authors mean by “…changes in HIVDR…” are they referring to changing trends in HIVDR across different antiretroviral drug classes? or variations by geographic location of the study participants? or differences based on the ART regimens participants are receiving?	2. Abstract: Page 2 (line 29); To improve clarity, we have removed “changes in” and replaced with “patterns of”
3. Lines 34 – 35 (Results section of the abstract): typically adolescence refers to the age range between 10 and 19 years, and childhood spans from 0 to 9 years. However, the authors reported an age range of 0 to 17 years… Was there no data available for individuals aged 18 and 19? Please provide justification for the chosen age range.	3. There was data available for individuals aged 18 and 19 years, which we did not include because it was previously published by our group. For further clarity, in Methods section, Page 6 (lines 128-129) we have rephrased the sentence as follows “aligning with our previous research on geospatial mapping of HIV-1 VLs [18] and HIVDR among individuals >17 years of age [19].”
4. What proportion of participants with NNRTI DRMs were actually on these antiretrovirals given that these mutations can persist for a long time due to their prolonged half-lives in the body?	4. Out of 609 participants with NNRTI DRMs conferring resistance, 32 were on NNRTI-based regimens i.e. 5.25%. We have rephrased the Abstracts results paragraph to include this in Page 2 (line 36-39); “Of the 740 with HIVDR, 609 (82.30%) have non-nucleoside reverse transcriptase inhibitor (NNRTI) mutations with 32/609 (5.25%) on NNRTIs; 525/740 (70.95%) have nucleoside reverse transcriptase inhibitor (NRTI) mutations, and 181/740 (24.46%) have major protease inhibitor (PI) mutations.”
5. Lines 42 to 43: It would be interesting to report the resistance levels to TDF as well, as this NRTI is the backbone of the currently recommended TLD (Tenofovir + Lamivudine + Dolutegravir) regimen.	5. Abstract: Page 2 (line 42-45); We have reported the resistance levels to TDF and rephrased the sentence as follows: “GIS-interpolation maps show potential-low-level resistance to tenofovir, low-to-intermediate-level resistance to abacavir (high-level in northern localities) and intermediate-to-high-level resistance to lamivudine, nevirapine and efavirenz.”
6. Introduction: Line 72: It is unclear why CALWH aged <3 years are specifically referred to as a composite group. Why	6. Introduction: Page 3 (line 74); We specifically referred to CALWH aged <3 years because the South African guidelines from 2013 to 2019

was this particular age group singled out, and how is this distinction relevant for the later analyses? Are these children not a distinct age category, separate from adolescents, rather than part of a combined population?	has a distinction in CALWH aged <3 years receiving AZT/3TC/LPV/r as first line ART, with EFV replacing LPV/r in older CALWH. We have described these guideline changes in Page 4 (lines 77-83).
7. Lines 75 and 97: Providing the exact number of CALWH, rather than simply stating "…largest HIV-1 epidemic…" or "… Given the high prevalence of HIVDR among pediatrics in South Africa…", respectively, would help readers better understand the magnitude and relevance of this population.	7. To help readers better understand the magnitude and relevance of this population, the proportion of CALWH has been provided in Page 4 (lines 97-98); "South Africa is home to approximately 10% of the global population of CALWH". The high prevalence of HIVDR has been provided in Page 4 (lines 96-97); "highest HIVDR prevalence of 37.80% [95% confidence interval (CI) 26.24-50.08] observed among ART-naïve children in Southern Africa"
8. Lines 98 to 99: The authors state that "…there is a critical need for effective near-real time geospatial surveillance of HIVDR in CALWH…," but this claim lacks adequate justification in the introduction section. This comes out of the blue in this section without prior clarification about what they mean by "effective near-real time geospatial surveillance", nor explanation about how this approach would integrate with or enhance existing HIVDR surveillance strategies. This statement would benefit from further clarification and contextual grounding, especially given that it addresses a core aspect explored in the manuscript.	8. Thank you for pointing this out. For further clarification, we have provided an explanation about how this approach would integrate with or enhance existing HIVDR surveillance strategies. Page 5 (lines 103-110); "Given the high prevalence of HIVDR among paediatrics in South Africa [3], there is a critical need for effective near-real time surveillance of HIVDR in CALWH. Geospatial analysis has the potential to be an effective surveillance technique for predicting, via interpolation approaches, areas with increased HIVDR in resource-limited settings. Utilizing these geo-localized analyses, policymakers can make more informed decisions regarding the allocation of healthcare workers to rural versus urban primary healthcare facilities or enhance specialized infectious disease services in regions with sustained high-level viraemia and HIVDR among treatment-experienced CALWH."
9. Materials and Methods: Paragraph 1: Could the authors specify how many public health facilities were included in the study?	9. Materials and Methods: We have specified how many public health facilities were included in the study. Page 6 (line 147); "healthcare facilities (n=114)"
10. Lines 115 to 117: Given that the authors report relatively high pretreatment drug resistance in the study population (lines 89 to 93), could they clarify the justification for waiting two years post-DTG initiation before GRT? A brief	10. We have added a sentence to indicate that this recommendation aligns with standard of care in South Africa. Page 6 (lines 139-140); "Essentially GRT is indicated in specific scenarios of virological failure according to standard of care in South Africa."

justification (if any) on how this recommendation aligns with current WHO or regional guidelines on HIVDR and balances risks and benefits in this specific context would strengthen the manuscript.	
11. Line 127: As already mentioned in an earlier comment in the abstract section, it is still unclear why the authors defined adolescence as ages 10 to 17 years instead of the more commonly used age range of 10 to 19 years, as indicated in the reference they provided. Please justify this choice.	11. There was data available for individuals aged 18 and 19 years, which we did not include because it was previously published by our group. For further clarity, in Methods section, Page 6 (lines 128-129), we have rephrased the sentence as follows "aligning with our previous research on geospatial mapping of HIV-1 VLs [18] and HIVDR among individuals >17 years of age"
12. Figure 1 presents key findings and is already part of the results; therefore, it should be moved to the results section and used to form the first paragraph of this section to ensure proper flow of information.	12. We have moved Figure 1 to the results section and used it to form the first paragraph of this section. Page 10 (lines 224-230).
13. Please include a brief description of the different ART regimens to which the participants included in the analysis were exposed. This information would improve understanding of the interpretation of GRT drug resistance mutations and enhance the public health relevance of the findings, especially in the context of limited availability of ARV drug formulations. This would also improve the understanding of the results provided in lines 193 to 196; lines 208 to 213.	13. We have included a brief description of the different ART regimens to which the participants included in the analysis were exposed. Page 7 (lines 167-170); "The combination of ARV drugs within ART regimens varied, therefore GRT records with complete regimen data (n=851) were categorized further based on the NRTI contained i.e. ABC, zidovudine (AZT) and TDF. Regimens included XTC (emtricitabine/lamivudine) and either efavirenz (EFV), PI/r, INSTI or PI/r plus INSTI."
14. Lines 168 to 174 (Statistical analysis): Please provide more information on the variable types including those used in the modelling; were continuous variables like viral loads modeled as continuous or categorized? Did you perform any adjustments or focused just on univariate analysis? The choice of a one-sided chi-square test is statistically unconventional, what was the rationale for this choice? How was missing data handled in your analysis?	14. We have provided more information on the variable types, statistical analysis and handling of missing data. Page 8-9 (lines 194-200); "We used descriptive statistics to assess patterns of HIVDR, GSSs and mutations, and presented the results as percentages in graphs and tables. To evaluate the associations between HIVDR and patient GRT variables, we analysed continuous variables i.e. VL and numerically converted categorical variables i.e. age group using logistic regression and estimated odds ratios with cluster-robust standard errors. Additionally, we analysed categorical variables (year of collection, sex

	and patient status) using Chi-square tests. GRT records with missing VL data were excluded from the logistic regression analysis."
15. Statistical analysis: - There is no need to present both proportions and percentages, presenting percentages alone would be clearer and more consistent.<br><br>- Additionally, the section on logistic regression modelling would benefit from more detail. For example, did the authors account for potential clustering by health facility or district? Were possible confounding factors considered when exploring factors associated with drug resistance?<br><br>- Finally, did the models adjust for time to account for changes in treatment regimens, drug availability, and other relevant programmatic shifts?<br><br>- Could the authors provide additional details regarding the geospatial analysis to enhance the clarity and reproducibility of the study?	15. We have revised the sentence. Page 8 (lines 194-195); "We used descriptive statistics to assess patterns of HIVDR, GSSs and mutations, and presented the results as percentages in graphs and tables."<br><br>We have added more details to the section on logistic regression modelling. Page 8 (lines 195-198); "To evaluate the associations between HIVDR and patient GRT variables, we analysed continuous variables i.e. VL and numerically converted categorical variables i.e. age group using logistic regression and estimated odds ratios with cluster-robust standard errors."<br><br>We have included this in the limitations section. Page 21 (lines 426-428) "We did not consider district/facility HIVDR testing practices, nor contextualize our geographical analysis within the local CALWH population, nor account for ART coverage by district, subdistrict or facility."<br><br>We have provided additional details regarding the geospatial analysis. Page 8 (lines 189-191); "Sensitivity analyses, conducted by adjusting the power exponent, demonstrated an ideal power of 2 for ARV drug susceptibility IDW map creation."<br><br>We have included a new section called "Statistics and Reproducibility". Page 9 (lines 202-209); "Statistics and Reproducibility<br>We conducted all statistical analyses in Stata 18.0 software SE (StataCorp. 2023, College Station, TX, USA) using Stata do-files to ensure reproducibility of results. NHLS CDW provided standardized data from routinely processed GRTs. Uniformly linked facility GIS information from 114 public-sector healthcare facilities served as unique geographic locality "replicates" which could be repeatedly

	geospatially analysed by year and district to determine the ARV drug susceptibility levels among CALWH in KZN. For GRTs exhibiting HIVDR, mutations were further categorized based on ARV drug class for cumulative analyses."
16. Results: The authors should include a clear description, early in the manuscript, of the characteristics of the CALWH for whom the 914 records were available, for example, their age distribution, sex, and other relevant demographics. This is essential to properly contextualize the subsequent risk factor analyses.	16. We have added a sentence on characteristics of CALWH. Page 9 (lines 221-223); "Patients had a median age of 13 years (Interquartile range (IQR): 8-16) and 49.34% (451/914) were female. Figure 1 presents a summary of the GRT records that were retrieved and analysed."
17. Lines 188 – 189: The sentence 'Notably, the proportion of HIVDR was significantly higher in children compared to adolescents (92.22% versus 76.56% respectively)…' presents percentages, as such, I suggest replacing 'proportion' with 'percentage' for accuracy.<br><br>- I also suggest removing 'Notably' as this implies interpretation of the results, whereas simply stating the facts directly would be clearer in this section.<br><br>- Additionally, please add the exact age ranges for 'children' and 'adolescents' to enhance clarity.	17. We have rephrased the sentence, removed "proportion and "Notably", and added the exact age ranges for 'children' and 'adolescents' to enhance clarity. Page 10 (lines 232-234); "HIVDR was significantly higher in children (0-9 years) compared to adolescents (10-17 years): 92.22% versus 76.56% respectively, p<0.05."
18. Lines 189 – 193: To enhance clarity and facilitate interpretation, I recommend including these results in a dedicated table. I suggest a Table 2 presenting the odds ratios along with clear information about the variables adjusted for.	18. We have included these results in a supplementary table to enhance clarity and have updated the reference to the supplementary table in the manuscript. Page 11 (line 237); "Supplementary Table S1".
19. Table 1: If available, please include details on ART treatment duration for the study participants. This information is critical for enhancing the understanding and interpretation of HIVDR, bearing in mind that HIVDR is	19. Unfortunately, data on ART treatment duration was not available. We have included this as a limitation. Page 21 (lines 421-423); "Data regarding previous ARV drug regimens, adherence and ART duration were not consistently documented, and we did not merge serial GRTs, if present, within the HIVDR database."

closely linked to cumulative drug pressure overtime, which drives the selection of drug resistance mutations.<br>- The label 'Year of collection' needs further clarification. Was there one record per individual for each time period, or multiple records over time?<br><br>- Additionally, it would be helpful if the authors could explain how they accounted for potential time-varying confounders, such as changes in age, in their analysis.<br><br>- Could the authors please clarify the rationale for the chosen age categorization in this table, especially since the authors report on the <3 years age group?<br><br>- Could the authors clarify the meaning of the label 'Inpatients' in table 1 to ensure readers fully understand the category?	For further clarification, we have added a footnote regarding year of collection to Table 1. Page 12 (lines 245-247); "[a]Year of collection refers to the year in which each genotypic resistance test (one record per patient) was requested. For year 2022, the data represents the first half of the year, as the study period concluded on 30 June 2022."<br><br>We have added this to the limitations. Page 21 (lines 423-424); "We did not account for potential time-varying confounders in the analysis, particularly, changes in patient age."<br><br>We have revised the age groups throughout the manuscript, as defined in the methods section, to ensure uniformity. Page 7 (lines 165-166); "We further categorized age into 4 groups: (i) 0-4 years, (ii) 5-9 years, (iii) 10-14 years and (iv) 15-17 years"<br><br>For further clarification, we have changed the label to "Inpatients/admissions" on Page 11 (Table 1) and Page 7 (line 164).
20. Please note that an odds ratio of 3 does not directly mean that the outcome is 'three times higher' in the same way a prevalence ratio does. This misinterpretation must be corrected to ensure accurate reporting of the results. Additionally, all odds ratios should be clearly presented in the tables, not just selectively reported in the text.	20. We have rephrased the sentence and included a table of odds ratios in Supplementary Table S1. Page 10-11 (lines 234-237); "When compared to adolescents aged 15-17 years, the odds of HIVDR was nearly 3 times higher in children aged 0-4 years (odds ratio (OR)=2.94, 95% confidence interval (CI): 1.54–5.60, p=0.001), and over 4 times higher in children aged 5-9 years (OR=4.15, 95% CI: 1.94–8.89, p<0.001) (Supplementary Table S1)."
21. Lines 231 to 233: The results on multiclass drug resistance would be clearer if the authors included a brief overview of the treatment guidelines recommended, as well as the treatment regimens available in the South African context.	21. We have provided a brief overview of the treatment guidelines and regimens available in South Africa. Page 4 (lines 83-86); "Subsequently, WHO and South African national guidelines recommended DTG-based regimens in all children >1 month of age and ≥3 kgs, with current first-line regimens: ABC+3TC+DTG (<30kgs) or TDF+3TC+DTG (≥30kgs), and protease inhibitors (PIs) reserved for individual cases where DTG is unsuitable"

22. Lines 242 – 246: "When comparing regimens containing…" Could the authors clarify or provide more details in the Methods section regarding the analysis mentioned here, as it is not currently described?	22. We have provided more detail in the Methods section under statistical analysis and included a table of odds ratios in Supplementary Table S1. Page 8 (lines 195-198); "To evaluate the associations between HIVDR and patient GRT variables, we analysed continuous variables i.e. VL and numerically converted categorical variables i.e. age group using logistic regression and estimated odds ratios with cluster-robust standard errors."
23. It would be helpful to provide more detail on the other risk factors considered in the analysis. I suggest including a clear section describing the logistic regression methods used, along with the full output of this modelling, to strengthen the interpretation of your results.	23. We have provided more detail in the Methods section under statistical analysis and included a table of odds ratios in Supplementary Table S1. Page 8 (lines 195-198); "To evaluate the associations between HIVDR and patient GRT variables, we analysed continuous variables i.e. VL and numerically converted categorical variables i.e. age group using logistic regression and estimated odds ratios with cluster-robust standard errors."
24. Current treatment guidelines recommend switching to DTG-based regimens for pediatric patients. Given this, it would be helpful if the authors could comment on why only 8 participants in the study were on DTG, and discuss any potential implications this may have for the findings..	24. We have expanded on why only 8 participants in the study were on DTG, in the discussion section. Page 20 (lines 407-410); "Despite the rollout of DTG in December 2019, there has been a lag in transitioning CALWH to DTG-containing regimens in South Africa. The National treatment guidelines were revised in 2023 to include dispersible DTG tablets for improved dosing in paediatric patients [11], however, the availability of these dispersible tablets remains limited."<br><br>We discuss any potential implications this may have for the findings under methods, and limitations.<br>Page 7 (lines 155-157) "Given that only a few GRTs (n=8) included IN genotyping, and to avoid bias, we did not conduct geospatial analysis of the integrase strand transfer inhibitor (INSTI)-resistance mutations."<br>Page 21 (lines 428-431); "Given that only 8 CALWH were on DTG-based regimens, we could not geospatially analyse drug susceptibility levels for DTG, nor could we conduct a stratified analysis comparing pre-DTG and post-DTG transition phases."

25. Discussion - Line 301: "…persistent viraemia…" this is challenging to ascertain because the authors did not report treatment duration or the proportions of participants on different antiretroviral drugs. Both of these are important to consider, given the varying genetic barriers of these drugs, and are necessary to fully interpret the resistance patterns observed, as some mutations arise more easily under specific drug pressure.	25. We have removed the sentence with the phrase "persistent viraemia" from the discussion section.<br><br>The actual treatment duration was not available, and we have stated this as a limitation. Page 21 (lines 421-422); "Data regarding previous ARV drug regimens, adherence and ART duration were not consistently documented"
26. Paragraph 1, lines 301 – 306: I would suggest to focus on providing a summary of the main findings here and to reserve interpretation and discussion of these findings for later in the section.	26. We have provided a summary of the main findings in the first paragraph of the Discussion. Page 18 (lines 342-355); "This retrospective study of 914 routine HIV GRTs from children and adolescents in KwaZulu-Natal, South Africa, showed that HIVDR was 3 to 4 times higher in GRT records from children aged 0-9 years when compared to adolescents aged 15-17 years. This is consistent with results from routine GRTs processed between November 2017 and December 2018, which indicated a higher prevalence of PI resistance mutations in children compared to adolescents (43% versus 26%) [25] in KZN. Geospatial analysis of all GRTs revealed that most districts across KZN showed low-level resistance to PI/r drugs despite 9 out of 10 CALWH receiving PI/r-based regimens, consistent with the well-established high genetic barrier to resistance associated with PIs. Overall, the temporal trends of dual- and triple-class resistance were identified in 3 out of every 5 children. These trends are concerning, given the limited number of ARV drug classes available for optimization of second- and third-line ART in South Africa. Notwithstanding the shift to more potent TDF- and DTG-based regimens from 2018 to 2022, the prevalence of resistance remained unchanged. Only 8 GRTs in this study were from CALWH on DTG-based ART, however 62.50% showed GSSs ≥2 indicating sustained efficacy of the regimen."

27. For lines 310–311: please clarify if the comparison between children under 3 years and those over 3 years was explicitly outlined in the methods section to ensure consistency.	27. To ensure consistency, we have outlined the comparison of age groups in the methods section. Page 7 (lines 165-167) “We further categorized age into 4 groups: (i) 0-4 years, (ii) 5-9 years, (iii) 10-14 years and (iv) 15-17 years, relative to the regimens that were used for paediatrics in South Africa during the study period”
28. It would strengthen the discussion to more thoroughly relate the findings to existing literature from other countries, providing a broader comparative context.	28. To strengthen the discussion, we have related the findings to existing literature from other countries and updated the references accordingly.<br>Reference 2: “Jordan, M. R. et al. Human Immunodeficiency Virus (HIV) Drug Resistance in African Infants and Young Children Newly Diagnosed with HIV: A Multicountry Analysis. Clin. Infect. Dis. 65, 2018–2025 (2017).”<br>Reference 16: “Ge, L. et al. Global, regional, and national prevalence of HIV-1 drug resistance in treatment-naive and treatment-experienced children and adolescents: a systematic review and meta-analysis. eClinicalMedicine 77, 102859 (2024).”<br>Reference 27: “Fokam, J. et al. HIV-1 Drug Resistance in Children and Implications for Pediatric Treatment Strategies: A Systematic Review and Meta-analysis. Open Forum Infect. Dis. 12, (2025).”
29. Consider including an analysis and discussion of results by sex, if possible, as this could provide valuable insights	29. An analysis of results by sex is included in Table 1 and in the results section, which showed no statistically significant difference in HIVDR between male and female groups. Page 10 (line 232) “There was no significant difference in proportion of HIVDR by sex.”
30. Minor comments : Abstract<br>The abstract could benefit from additional details to enhance clarity for the reader.<br>- For example, it would be helpful to specify the time period over which trends were assessed, the number of facilities included, the type of genotypic resistance testing (GRT) performed, and the kind of GIS analysis conducted.	30. Abstract: The time period over which trends were assessed, the number of facilities included, the type of genotypic resistance testing (GRT) performed, and the kind of GIS analysis conducted have been included in the abstract. Median age of the 914 CALWH has been included. The methods now include logistic regression.<br>Page 2 (lines 31-34); “We analysed 914 routine HIV-1 genotypic resistance tests (GRTs) obtained between January 2018 and June 2022 from 0–17-year-old CALWH, integrating facility-linked Geographic Information Systems (GIS) coordinates for geospatial, descriptive and logistic regression analyses.”

- Additionally, in the results section of the abstract, please consider indicating the age range or median age of the 914 CALHW included.<br><br>- Finally, since an odds ratio is presented in the abstract, it would be important to clarify in the methods whether a logistic regression was conducted and, if so, to briefly describe it.	Page 2 (lines 35-36); "Here we show that 80.96% (740/914) of GRTs from CALWH (median age 13 years) across 114 facilities demonstrate HIVDR."<br>Page 2 (lines 42-45); "GIS-interpolation maps show potential-low-level resistance to tenofovir, low-to-intermediate-level resistance to abacavir (high-level in northern localities) and intermediate-to-high-level resistance to lamivudine, nevirapine and efavirenz."
31. The abstract mentions GIS mapping in the results section, but it does not specify which locations in KwaZulu-Natal (KZN) had higher or lower levels. Adding more details here would strengthen the conclusion, which currently emphasizes the potential of geospatial analysis for data-driven public health decision-making but lacks specific geographic context.	31. We have added more detail to strengthen the conclusion.<br>Page 2 (lines 46-49); "Routine GRTs demonstrate higher HIVDR prevalence among younger children, while ART susceptibility maps show overall uniformity with variations in certain regions, such as northern KwaZulu-Natal localities. GIS-linked real-time monitoring of HIVDR is crucial to assess the impact of the revised HIV-1 treatment guidelines for CALWH in South Africa."
32. Plain language summary: Line 56: When the authors mention "… younger children…", which specific age group are they referring to? Please specify this age group in brackets for clarity.	32. We have specified the age group for clarity. Page 3 (lines 57-58); "We find that children failing ART aged 0-9 years have higher HIVDR prevalence compared to those aged 10-17 years."
33. Line 58: Specify the age group in brackets here as well for clarity.	33. We have specified the age group for clarity. Page 3 (lines 57-58); "We find that children failing ART aged 0-9 years have higher HIVDR prevalence compared to those aged 10-17 years."
34. Materials and Methods: Study population and design: Line 107: Please specify the study design… for example, is it a cohort study?	34. We have specified the study design. Page 6 (line 124); "This retrospective cross-sectional observational study analysed routine HIV-1 GRT records"
35. Line 110: Did the authors conduct a stratified analysis comparing the pre-transition and transition phases to DTG, and the effect this transition had on the prevalence of HIVDR?	35. We did not conduct a stratified analysis, because only 8 GRTs in this study were from CALWH on DTG-based ART due to the lag in transition to DTG-containing regimens in South Africa. We have added this to the limitations. Page 21 (lines 428-431); "Given that only 8 CALWH were on DTG-based regimens, we could not geospatially analyse drug susceptibility levels for DTG, nor could we conduct a

	stratified analysis comparing pre-DTG and post-DTG transition phases.”
36. Results: Table 1: With median viral loads this high, did the study assess adherence to treatment in these participants? Major mutations being mostly quasi-species mutations may further indicate adherence challenges (lines 210 to 212).	36. The study did not assess adherence to treatment in these participants. We have added this to the limitations. Page 21 (lines 421-423); “Data regarding previous ARV drug regimens, adherence and ART duration were not consistently documented, and we did not merge serial GRTs, if present, within the HIVDR database.”
37. For clarity and consistency, I suggest reporting percentages to one decimal place and presenting p-values in brackets.	37. We have reported the percentages to 2 decimal places for accuracy, because rounding off to 1 decimal place results in a total >100%. As suggested, we have presenting p-values in brackets. Page 11 (Table 1).
38. Table 1: It may be helpful to remove the column header ‘No HIVDR’ as this is already implied by the ‘HIVDR’ column. Additionally, please clarify which statistical test was used to generate the p-values shown, as this information would help readers interpret the results more easily.	38. We have removed the column header ‘No HIVDR’ as suggested. Page 11 (Table 1). To clarify which statistical test was used to generate the p-values shown, we have added a footnote. Page 12 (lines 249-251); “P-values for year of collection, sex and patient status were generated using Chi-square tests while p-values for age group and viral load were generated using logistic regression with cluster-robust standard errors. Bold p-values indicate statistically significant results.”
39. Discussion: Lines 298 to 301: “… Comparable results… in KZN.” Please rephrase this sentence for clarity, as the purpose and meaning of the comparison are not clearly conveyed.	39. We have rephrased the sentence for clarity. Page 18 (lines 344-347); “This is consistent with results from routine GRTs processed between November 2017 and December 2018, which indicated a higher prevalence of PI resistance mutations in children compared to adolescents (43% versus 26%) [25] in KZN”
40. Line 308: Please adjust this to “HIVDR was predominantly driven by NNRTI mutations,…” Additionally, do the South African guidelines still recommend NNRTIs or have these been phased out in the current recommendations?	40. We have rephrased the sentence for clarity. Page 18 (line 358); “HIVDR was predominantly driven by NNRTI mutations” Additionally, the South African guidelines do not recommend NNRTIs in the current recommendations. We have included the current recommendations in the Introduction section. Page 4 (lines 83-86); “Subsequently, WHO and South African national guidelines recommended DTG-based regimens in all children >1 month of age and ≥3 kgs, with current first-line regimens: ABC+3TC+DTG (<30kgs) or TDF+3TC+DTG (≥30kgs), and protease inhibitors (PIs) reserved for individual cases where DTG is unsuitable”

41. Line 310: “When comparing children less than 3 years…”	41. For consistency, we have used the same age groups throughout the manuscript. Page 19 (lines 364-367); “When we compared children ≤4 years to those >4 years in our study, the prevalence of NNRTI resistance was 69% versus 66%, respectively. Of children ≤4 years, 61% had no present exposure to NNRTI drugs as part of their regimen.”
42. Line 328: the phrase “These geospatial analyses maps…” could be simplified by removing the word “maps” for clarity.	42. We have rephrased the sentence for clarity. Page 19 (lines 379-381); “These geospatial analyses demonstrated variations in ARV drug susceptibility levels, emphasizing the need for enhanced HIV-1 treatment monitoring in CALWH.”
43. There is repetition of results in the discussion section; it would be beneficial to focus more on comparing and contrasting these findings with those from other studies to highlight their significance.	43. We have removed certain repetitive sentences from the discussion section and have restructured the discussion to focus on comparing these findings with those from other studies to highlight their significance. Pages 18-22 (lines 341-438).
44. General comment: There are too many acronyms used throughout the manuscript, including in the abstract. I suggest considering reducing the number of acronyms where possible to enhance readability and make the text more accessible for a broader audience.	44. To enhance readability, where possible, we have removed certain “uncommon” acronyms. Page 2 (lines 29, 30, 48); Rewrote “KZN” as “KwaZulu-Natal”

Reviewer 3
The statistical analysis is well explained and appropriate for the study.

Response to Reviewer 3
Thank you for reviewing the manuscript.

Reviewer 4
General Comments

Summary
In the article entitled “HIV-1 drug resistance among ART-experienced children and adolescents in KwaZulu-Natal, South Africa: Geospatial analysis” Gounder et al have assessed the prevalence and geospatial trends of HIV drug resistance (HIVDR) mutations amongst the children

and adolescents living with HIV (CALWH) in the KwaZulu-Natal region of South Africa between 2018-2022. The authors have found the HIVDR to be more prevalent in children (0-9 years) compared to adolescents (15-17 years) with sustained virological burden. Higher levels of HIVDR to lamivudine, nevirapine and efavirenz were observed based on the geospatial analysis across KZN province. The article has highlighted the importance of real time geospatial trend analysis for effective targeted programmatic interventions in the hyperendemic regions. This has relevance not only for south Africa with highest burden of HIV but also globally, with the HIV pandemic now being driven through specific transmission clusters amongst high-risk populations.<br><br>Overall impression of the work<br>The article is well written, largely descriptive in nature and includes comprehensive statistical analyses of HIVDR, HIVDR distribution trends across geographical locations, age groups and genders. The data provided is however limited by the availability of comprehensive case information in the resource limited setting of KZN. The authors have included and addressed most of the confounders in the discussion section adequately. As discussed by the authors, the inconsistencies in refering cases for GRT in spite of high viral load (>1000 copies/mL) is a major limitation of this work that limits its impact.<br><br>Specific comments, with recommendations for addressing each comment *(see below)*<br><br>**Response to Reviewer 4**<br>Thank you for reviewing the manuscript and for the detailed comments. We have made the following changes to the manuscript:	
**Specific Comments**	**Response**
1. It is unclear what the overall burden of CALWH at each of the 11 districts in the KZN region is. Based on the supplementary data provided the range of GRT data varies between 15 (Harry Gwala) to 324 (eThekwini), cumulatively over the study period of 5 years. This would be important to delineate whether the included cases are proportionately representative of the geospatial trends. If not, this needs to be included and discussed as a potential limitation of the study with inclusion of district-wise distribution of cases.	1. We have discussed this as a potential limitation of the study. Page 21 (lines 426-428); "We did not consider district/facility HIVDR testing practices, nor contextualize our geographical analysis within the local CALWH population, nor account for ART coverage by district, subdistrict or facility."
2. The authors should clarify if any longitudinal sampling was considered from any of the study participants.	2. Longitudinal sampling was not done, and we have included this as a limitation. Page 21 (lines 422-423); "we did not merge serial GRTs, if present, within the HIVDR database." We have also added further clarity on study design to the methods section. Page 6 (line 124); "This

	retrospective cross-sectional observational study analysed routine HIV-1 GRT records”
3. The authors have described the participant grouping to be as follows (line 151), (i) 0-3 years, (ii) 4-9 years, (iii) 10-14 years and (iv) 15-17 years. The authors have also consistently differentiated the observations related to the 0-3 age group from the others. However, Table 1 with statistical analyses refers to different age groups. Uniform grouping would make the trends clearer.	3. As suggested, we have used uniform age grouping throughout the manuscript to make the trends clearer. We have defined the age groups in the methods section. Page 7 (lines 165-167); “We further categorized age into 4 groups: (i) 0-4 years, (ii) 5-9 years, (iii) 10-14 years and (iv) 15-17 years, relative to the regimens that were used for paediatrics in South Africa during the study period”
4. The article would benefit from further elaboration on the genotypic susceptibility scores section (lines 154-158) preferably with an example. Are the scores included for only the drugs taken by the participants or all of the drug classes as predicted by the Stanford HIV database? This isn’t clear in the supplementary files.	4. We have elaborated further on the genotypic susceptibility scores section and have added an example. The scores included are for the drugs taken by the participants. Page 8 (lines 176-180); “For example, if the patient is taking ABC+3TC+DTG and a GRT indicates the presence of K65R and M184V mutations, these confer high-level resistance (GSS=0) to ABC and 3TC, respectively. Therefore, the total GSS for ABC+3TC+DTG (0+0+1) is 1 i.e. <2 indicating reduced potency of the regimen for that patient.”
5. Utility of geospatial trend analysis for targeted interventions is logical. The authors have discussed dispersed sections of northern KZN districts having higher abacavir resistance prevalence (lines 325-327). However, the presented case does not seem convincingly in support of the geospatial analysis. The authors may discuss the potential explanation for this observation and how it can be addressed to make it more impactful.	5. We have rephrased the sentences related to abacavir geospatial analysis in the results and discussion sections. Page 16 (lines 321-322); “Furthermore, most districts showed low-level to intermediate resistance to ABC, however five out of the six localized areas of high-level resistance were observed in northern KZN.”<br>Page 19 (lines 374-377); “Five out of the six notably dispersed localities of high-level resistance to ABC were found in northern KZN districts, suggesting the need for targeted programmatic interventions in this region which is historically known for HIV-1 hyperendemicity [30–32].”
**Reviewer 5**<br>**General Comments**<br><br>REVIEW REPORT<br>Review_summary<br>Review summary<br>English language	

( ) I am not qualified to assess the quality of English in this paper
( ) English very difficult to understand/incomprehensible
( ) Extensive editing of English language required
( ) Moderate editing of English language required
(X) Minor editing of English language required
( ) English language fine. No issues detected

COMMENTS
The draft manuscript by Gounder et al, entitled "HIV-1 drug resistance among ART-experienced children and adolescents in KwaZulu-Natal, South Africa: Geospatial analysis" addresses a critical issue of HIVDR among children and adolescents in South Africa. The analysis has been done with a large dataset of 914 GRTs among CALWH in a hyperendemic setting. The integration of geospatial analysis is particularly innovative and an inspiration for health researchers in the region. The study highlights a concerningly higher prevalence of HIVDR among CALWH across KZN. These useful findings are crucial in informing policy not just within South Africa but in similar settings. The study also highlights interesting policy gaps in the monitoring of HIVDR among CALWH.

SUGGESTIONS FOR IMPROVEMENT
I would like to highlight the following observations to the authors. *(see below)*

Recommendation
This draft manuscript is interesting and, in my opinion, worthy of publication. It would, however, require moderate revision to address the issues I have suggested above.

Response to Reviewer 5
Thank you for reviewing the manuscript and for the detailed comments. We have made the following changes to the manuscript:

**Specific Comments**	**Response**
1. While the title is clear and concise, I I suggest amending it to, "….: A geospatial analysis" to improve readability	1. We have amended the title as suggested. Page 1, (lines 1-2); "Geospatial analysis reveals similar HIV-1 drug resistance patterns among ART-experienced children and adolescents of South Africa"
2. In the abstract, I suggest highlighting the sample size in the methods section. E.g. We analyzed 914…	2. We have highlighted the sample size in the abstract methods section as suggested. Page 2 (lines 31-34); "We analysed 914 routine HIV-1 genotypic resistance tests (GRTs) obtained between January 2018 and June 2022 from 0–17-year-old CALWH, integrating facility-linked Geographic

	Information Systems (GIS) coordinates for geospatial, descriptive and logistic regression analyses."
3. In line 45, "general uniformity" could be modified to read as "overall uniformity with variations in regions"	3. We have modified the sentence as suggested. Page 2 (lines 46-48); "Routine GRTs demonstrate higher HIVDR prevalence among younger children, while ART susceptibility maps show overall uniformity with variations in certain regions, such as northern KwaZulu-Natal localities."
4. The statement in lines 66-70 could be shortened to improve readability.	4. We have shortened the statement by splitting it into 2 sentences. Page 3 (lines 68-72);<br>"Children and adolescents living with HIV (CALWH) in resource-limited settings face an increased risk of developing HIVDR."<br>"This is due to limited child-friendly drug formulations, adherence challenges, dose adjustments, maternally transmitted HIVDR and potential prior exposure to non-nucleoside reverse transcriptase inhibitor (NNRTI)–based ART for prevention of vertical transmission (PVT)"
5. The authors highlight that South Africa is home to the largest pediatric HIV epidemic globally. This could be backed by current statistics and reference (s).	5. The current statistics and reference (s) have been provided in Page 4 (lines 97-98); "South Africa is home to approximately 10% of the global population of CALWH". The high prevalence of HIVDR has been provided in Page 4 (lines 96-97); "highest HIVDR prevalence of 37.80% [95% confidence interval (CI) 26.24-50.08] observed among ART-naïve children in Southern Africa"
6. In the methods section, I commend the authors for clearly describing the study design. However, I would suggest inclusion of subheadings such as study population, laboratory methods, geospatial mapping, to improve readability.	6. In the methods section, we have included subheadings.<br>Page 5 (line 123); "Study population and design"<br>Page 6 (line 145); "HIV-1 genotypic data analysis"<br>Page 7 (line 159); "HIVDR database curation and genotypic susceptibility scores"<br>Page 8 (line 182); "HIVDR geospatial analysis"<br>Page 8 (line 193); "Statistical analysis"<br>Page 9 (line 202); "Statistics and Reproducibility"<br>Page 9 (line 211); "Ethical considerations"
7. GSS is a technical concept that most non-specialist readers may find difficult to understand. I suggest authors describe what it is in a simplified language in the methods section.	7. In the methods, we have elaborated further on the genotypic susceptibility scores section and have added an example. The scores included are for the drugs taken by the participants. Page 8 (lines 176-180);

	"For example, if the patient is taking ABC+3TC+DTG and a GRT indicates the presence of K65R and M184V mutations, these confer high-level resistance (GSS=0) to ABC and 3TC, respectively. Therefore, the total GSS for ABC+3TC+DTG (0+0+1) is 1 i.e. <2 indicating reduced potency of the regimen for that patient."
8. The legends seem to be in the same font size as the rest of the text, and it may be difficult for readers to distinguish. I suggest modifying the texts of legends to distinguish them from the main text of the draft manuscript.	8. We have modified the texts of legends to distinguish them from the main text of the draft manuscript.<br>Page 10 (lines 226-230); Page 12 (lines 245-253); Page 13 (lines 264-270); Page 15 (lines 302-308); Page 17-18 (lines 329-339)
9. While the results appear with well-structured tables and figures, I suggest the following improvements:<br><br>i) In figure 2, the word major classes of surveillance drug resistance mutations (SDRMs) should be included in the horizontal axis.<br><br>ii) The line(s) at the horizontal axis of figure 2, indicating where PI, NNRTI, and NRTI end and or begin looks like a continuous line. I suggest it is redesigned to show distinction between the SDRMs.<br><br>iii) Authors should revise the results section and move statements of interpretation of results to the discussion section, instead. E.g. line 263… "Lower GSSs in patients on EFV containing regimens are largely due to NNRTIs low genetic barrier to resistance"	9. We have modified the figures and manuscript texts as suggested.<br><br>i) In figure 2, "Major classes of surveillance drug resistance mutations" has been included in the horizontal axis.<br><br>ii) To show distinction between the SDRMs, figure 2 has been redesigned. The lines at the horizontal axis have been changed to brackets and the graph bars have been coloured according to the major classes of SDRMs.<br><br>iii) We have revised the results section and moved statements of interpretation of results and linked references to the discussion section. Page 19 (lines 360-361) "Four out of every five patients on EFV-containing regimens had total GSSs <2 indicating reduced regimen potency, largely due to NNRTIs having a low genetic barrier to resistance[26]"
10. In the discussion section, the authors thoroughly interpreted the results and included relevant prior studies. The authors highlight clinical implications such as the need for real-time HIVDR surveillance. However, I suggest the following for improvement:	10. We have revised the discussion to encompass main findings, comparative studies, explanations and implications. To make the discussion structure clearer and to avoid the discussion appearing like a blend of results and literature review, we have included subheadings.

i) A clear structure be followed in the discussion to encompass main findings, comparative studies, explanations and implications.<br><br>ii) The narrative be streamlined to avoid the discussion appearing like a blend of results and literature review. Focus should be given to policy and clinical implications.<br><br>iii) Some sentences are too lengthy and could be revised to improve readability.	Furthermore, we have shortened/revised some lengthy sentences to improve readability.<br><br>Page 18 (lines 350-352); "Overall, the temporal trends of dual- and triple-class resistance were identified in 3 out of every 5 children. These trends are concerning, given the limited number of ARV drug classes available for optimization of second- and third-line ART in South Africa."<br><br>Page 19 (lines 367-370); "Several South African studies have highlighted the increasing prevalence of HIVDR in CALWH, primarily due to maternally transferred NNRTI-associated resistance [3,16,28,29]. These studies emphasize the urgent need for continued monitoring of this phenomenon as maternal ART guidelines transition to a DTG-based regimen [3,16,28,29]."
11. There is a need to proof-read the whole manuscript to eliminate typos e.g. line 349…due [to] poor adherence…	11. Thank you for pointing this out. We have proof-read the manuscript and have rephrased the sentence on Page 20 (line 399); "due to poor adherence"
12. In the conclusion section, line 389, the authors used the word 'significantly' without quoting the statistical p value. I suggest that this be revised. E.g. "By integrating routine HIV-1 GRT records with GIS analysis, we identified significantly (remarkably/ concerningly) higher levels of HIVDR among younger children.	12. We have rephrased the sentence on Page 22 (line 441-442); "By integrating routine HIV-1 GRT records with GIS analysis, we identified concerningly higher levels of HIVDR among younger children."

RESPONSE TO REVIEWER COMMENTS

Geospatial analysis reveals similar HIV-1 drug resistance patterns among antiretroviral therapy-experienced children and adolescents (0-17 years) of South Africa

**Reviewer 1 (Remarks to the Author):**<br>I co-reviewed this manuscript with one of the reviewers who provided the listed reports. This is part of the Communications Medicine initiative to facilitate training in peer review and to provide appropriate recognition for Early Career Researchers who co-review manuscripts.<br><br>**Response to Reviewer 1**<br>Thank you for reviewing the manuscript.

Reviewer 2 (Remarks to the Author):

Thank you for the opportunity to review the revised version of this manuscript. I appreciate the authors' efforts in addressing many of the comments raised during the first round of review. The revisions have improved several aspects of the manuscript, particularly the clarification of the study population, the use of terminology related to HIV drug resistance, and the specification of the antiretroviral regimens to which participants were exposed. The authors have also provided additional detail on the national treatment guidelines and made efforts to improve the clarity of the methodology and the overall presentation of the study. The geospatial component has likewise been strengthened, although some aspects still require further clarification.

The topic remains relevant, as the manuscript explores the use of geospatial approaches to better understand patterns of HIV-1 drug resistance (HIVDR) among children and adolescents living with HIV in a high-burden, resource-limited setting. The analysis of genotypic resistance data is thorough and provides valuable information on resistance patterns and prior treatment exposure in this vulnerable population.

However, the added value of the geospatial component is less clear. While mapping resistance patterns is a promising approach in principle, the manuscript does not yet fully demonstrate how this integration meaningfully strengthens surveillance or leads to actionable implications for treatment strategies in this context. Further clarification is needed on how the spatial analysis contributes beyond descriptive mapping and how it could realistically inform programmatic decision-making in similar settings.

While the revisions have improved the manuscript, several points would still benefit from clarification and adjustments to enhance overall clarity and consistency. These are outlined below for the authors' consideration. Overall, I would support acceptance after these revisions. In particular, the authors should clarify the central focus of the manuscript: either emphasize the strong genotypic resistance analysis with the geospatial component as supportive, or further strengthen the geospatial analysis and clearly demonstrate its added value for surveillance and treatment strategies, as currently suggested.

Response to Reviewer 2

Thank you for reviewing the manuscript and for the detailed comments. We have made point-by-point changes to the manuscript.

**Specific Comments**	**Response**

1. Further clarification is needed on how the spatial analysis contributes beyond descriptive mapping and how it could realistically inform programmatic decision-making in similar settings.<br><br>In particular, the authors should clarify the central focus of the manuscript: either emphasize the strong genotypic resistance analysis with the geospatial component as supportive, or further strengthen the geospatial analysis and clearly demonstrate its added value for surveillance and treatment strategies, as currently suggested.	1. For further clarification on how the spatial analysis contributes beyond descriptive mapping and how it could realistically inform programmatic decision-making in similar settings, we have included the following sentences:<br><br>Page 5 (lines 107-119); "Beyond descriptive mapping, geospatial analysis of antiretroviral (ARV) drug susceptibility levels can reveal localities of high-level resistance, even within regions where HIVDR appears uniformly distributed. Such geospatial analyses promote targeted and optimized changes to existing treatment programmes, enabling timeous test requests for linked ARV drug-levels and GRTs, providing earlier detection of HIVDR in CALWH. Combining geospatial analyses with health statistics, mobile network data, and environmental data, allow for the prediction of HIV burden estimates and new infections, with the future possibility of detecting at-risk geospatial localities ahead of traditional surveillance systems [18,19]. Further integration of the geospatial-health statistics model with machine learning and explainable artificial intelligence may enable, for example, the identification of appropriate candidates for pre-exposure prophylaxis within a defined hyperendemic region [18,19]."<br><br>Page 19 (lines 369-373); "Even in seemingly uniform regions, geospatial analysis of HIV GRT data can uncover localized areas of high-level resistance to ARV drugs. Spatial analyses can inform, for example, the provision of on-site training to healthcare workers within high-level resistance localities to reduce referral of patients to specialist healthcare facilities for timely HIV-1 GRT and ART regimen switching."
2. Title: The abbreviation ART appears in the title but is not defined. Consider spelling it out as antiretroviral therapy (ART) to improve clarity and comply with journal conventions regarding abbreviations.	2. To improve clarity and comply with journal conventions regarding abbreviations, we have amended the title as follows:<br>Page 1 (lines 1-2); "Geospatial analysis reveals similar HIV-1 drug resistance patterns among antiretroviral therapy-experienced children and adolescents (0-17 years) of South Africa"

	For consistency, we have also made the following changes:<br>Page 4 (line 88); Changed “programs” to “programmes”<br>Page 6 (line 127); Changed “antiretroviral” to “ARV”<br>Page 7 (line 162); Changed “antiretroviral (ARV)” to “ARV”<br>Page 22 (line 436); Changed “antiretroviral” to “ARV”
3. Line 40 (Results section of the abstract) – report the interquartile range (IQR) of the median age of the study participants.	3. We have rewritten the sentence to include interquartile range (IQR):<br>Page 1 (lines 35-36); “Here we show that 80.96% (740/914) of GRTs from CALWH (median age 13, interquartile range 7-15 years) across 114 facilities demonstrate HIVDR.”
4. Lines 40 – 42 (Results section of the abstract) – the sentence “Of the 740 with HIVDR, 609 (82.30%) have non-nucleoside reverse transcriptase inhibitor (NNRTI) mutations with 32/609 (5.25%) on NNRTIs…” is somewhat difficult to follow. Restructure this sentence for improved clarity and readability. In addition, please ensure that the appropriate past tense is used when reporting results.	4. To improve clarity and readability, we have restructured the sentence as follows:<br><br>Page 2 (lines 36-40); “Of the 740 individuals with HIVDR, 609 (82.30%) had non-nucleoside reverse transcriptase inhibitor (NNRTI) mutations, and among them, 32 (5.25%) were on NNRTI treatment. Additionally, 525 (70.95%) had nucleoside reverse transcriptase inhibitor (NRTI) mutations, and 181 (24.46%) had major protease inhibitor (PI) mutations.”
5. Line 52 (Conclusions section of the abstract) – the phrase “… demonstrate higher…” would benefit from clarification. Specify higher compared to what reference group or comparison ?	5. For clarity we have rephrased the sentence as follows:<br><br>Page 2 (line 47); “Routine GRTs demonstrate high HIVDR prevalence among younger children”<br><br>Page 21 (line 429-430); “By integrating routine HIV-1 GRT records with GIS analysis, we identified concerningly high levels of HIVDR among younger children.”
6. Introduction: the manuscript highlights a “crucial” or “urgent” need for GIS-linked HIVDR monitoring. While the authors attempt to clarify this point, the justification could be strengthened further. The introduction presents a compelling rationale for improving HIVDR surveillance among this vulnerable population; however,	6. We have expanded the Introduction section to briefly discuss the current state of the art in GIS applications for HIVDR or infectious disease surveillance, and have cited 2 relevant studies (new references 18 and 19):<br><br>Page 5 (lines 107-119); “Beyond descriptive mapping, geospatial analysis of antiretroviral (ARV) drug susceptibility levels can reveal localities of

it remains somewhat unclear how GIS-based approaches specifically enhance current monitoring strategies. Expanding this section to briefly discuss the current state of the art in GIS applications for HIVDR or infectious disease surveillance, and citing relevant studies where similar approaches have been used, would help better substantiate the proposed value of GIS-linked monitoring in this context. At present, this is not sufficiently addressed in the manuscript and would benefit from further development.	high-level resistance, even within regions where HIVDR appears uniformly distributed. Such geospatial analyses promote targeted and optimized changes to existing treatment programmes, enabling timeous test requests for linked ARV drug-levels and GRTs, providing earlier detection of HIVDR in CALWH. Combining geospatial analyses with health statistics, mobile network data, and environmental data, allow for the prediction of HIV burden estimates and new infections, with the future possibility of detecting at-risk geospatial localities ahead of traditional surveillance systems [18,19]. Further integration of the geospatial-health statistics model with machine learning and explainable artificial intelligence may enable, for example, the identification of appropriate candidates for pre-exposure prophylaxis within a defined hyperendemic region [18,19]. Ultimately,"
Additional comments (remaining issues from previous review round):<br><br>7. Abstract (Lines 29-30): In the previous review, I requested clarification on what was meant by "changes in HIVDR." The authors have replaced this with "patterns of," which improves wording but does not fully resolve the underlying ambiguity. It remains unclear whether this refers to variation by drug class, geographic location, or ART regimen. Please specify what "patterns" refers to in this context.	 <br><br>7. We have rephrased the sentence to say:<br><br>Page 2 (lines 29-30); "This study describes trends in HIVDR and patterns by drug class and geographic location among ART-experienced CALWH in KwaZulu-Natal."
8. Title: The inclusion of the age (children and adolescents) in the title was suggested in the previous round of review but has not been addressed. Given that the study population is specific and central to the manuscript, the age of the population included should be reflected in the title.	8. To improve clarity, we have amended the title as follows:<br><br>Page 1 (lines 1-2); "Geospatial analysis reveals similar HIV-1 drug resistance patterns among antiretroviral therapy-experienced children and adolescents (0-17 years) of South Africa"
9. Statistical analysis (Lines 193-200): The statistical analysis section remains limited in detail. In particular,	9. To improve clarity, we have rephrased the statistical analysis section as follows:

the authors should specify what clusters were used (i.e. was it the health facilities) for the cluster-robust standard errors. In addition, the sentence describing the logistic regression analysis (lines 195 - 199) would benefit from rephrasing for clarity. As currently written, it is difficult to follow, particularly regarding how continuous and categorical variables were handled and incorporated into the models. Clarifying the analytical approach would improve transparency and reproducibility.<br><br>Overall, I commend the authors for the revisions made in response to the previous review comments. Addressing the points above would further improve the clarity and contextual framing of the manuscript.	Page 1 (lines 207-212); "To evaluate the associations between HIVDR and patient GRT variables, we analysed continuous variables (i.e., VL and age), estimating odds ratios with cluster-robust standard errors. Standard errors were clustered at the healthcare facility level. Categorical variables (i.e., year of collection, sex and patient status) were analysed using chi-square tests. GRT records with missing VL data were excluded from the logistic regression analysis."<br><br>Thank you for reviewing the manuscript and for the detailed comments.

Reviewer 4 (Remarks to the Author):

The article entitled "HIV-1 drug resistance among ART-experienced children and adolescents in KwaZulu-Natal, South Africa: Geospatial analysis" reports on trends of higher HIVDR in younger CALWH in KwaZulu-Natal, South Africa. The study suffers from inherent limitations related to unequal sampling, reporting of HIVDR cases as well as lack of some key data. However, through the revised manuscript these have now been adequately indicated and discussed.

Response to Reviewer 4

Thank you for reviewing the manuscript and for the comments.

Reviewer 5 (Remarks to the Author):

I commend other reviewers for the comments they raised that have consequently improved the manuscript remarkably. I want to thank the reviewers for dedicating time to revise the manuscript.

The authors have satisfactorily addressed the comments I raised earlier.

Response to Reviewer 5

Thank you for reviewing the manuscript and for the comments. We have made the following changes to the manuscript:

**Specific Comments**	**Response**
1. The discussion has been revised thoroughly, and ideas are well-articulated. However, in the revised manuscript, I noticed	1. We have deleted the subheadings to allow a natural flow of the discussion.

that the discussion has subheadings. While I suggested a well-structured flow of ideas in the discussion, I would not recommend subheadings within the discussion. I suggest the authors delete these subheadings to allow a natural flow of the discussion.	
