# Supplementary material for: Geospatial analysis reveals similar HIV-1 drug resistance patterns among antiretroviral therapy-experienced children and adolescents (0-17 years) of South Africa
Source: Commun Med (Lond). 2026 Jul 7;6:570. doi: 10.1038/s43856-026-01770-9 (PMC13720034; doi:10.1038/s43856-026-01770-9)
Supplement: Supplementary file 2 [file 43856_2026_1770_MOESM2_ESM.pdf]

Supplementary Information

Geospatial analysis reveals similar HIV-1 drug resistance patterns among antiretroviral therapy-experienced children and adolescents (0-17 years) of South Africa

Lilishia Gounder [1,2*], Moherndran Archary [3], Aabida Khan [1,2], Kerusha Govender [1], Raveen Parboosing [1,4], Kogieleum Naidoo [5,6], Benjamin Chimukangara[1,5,7]

[1]Department of Virology, School of Medicine, University of KwaZulu-Natal, Durban, 4001, South Africa
[2]Department of Virology, National Health Laboratory Service, Inkosi Albert Luthuli Central Hospital, Durban, 4001, South Africa
[3]Department of Paediatrics, University of KwaZulu-Natal, Durban, 4001, South Africa
[4]School of Pathology, University of Witwatersrand & National Health Laboratory Service, Johannesburg, 2000, South Africa
[5]Centre for the AIDS Programme of Research in South Africa (CAPRISA), Durban, 4001, South Africa
[6]South African Medical Research Council (SAMRC), CAPRISA HIV-TB Pathogenesis and Treatment Research Unit, Durban, 4001, South Africa
[7]Critical Care Medicine Department, NIH Clinical Center, Bethesda, Maryland, 20892, United States of America

***Corresponding author**
Lilishia Gounder
Address: 800 Vusi Mzimela Road, Durban, 4051, South Africa
Tel: +27312402794; Email: gounder@ukzn.ac.za

Content Summary

This file contains the following supplementary material:

- Supplementary Table S1: Logistic regression models for HIV-1 drug resistance versus age group, drug regimen and viral load.
- Supplementary Table S2: HIV-1 drug resistance mutations detected in genotypic resistance tests from children and adolescents in KZN, South Africa.
- Supplementary Table S3: Antiretroviral drug regimens with genotypic susceptibility scores for children and adolescents from public sector healthcare facilities in KwaZulu-Natal, South Africa.
- Supplementary Figure S1: Temporal changes in the proportions of drug-class resistance observed in children and adolescents from KwaZulu-Natal, South Africa.

Supplementary Table S1. Logistic regression models for HIV-1 drug resistance versus age group, drug regimen and viral load

a) Model for HIV-1 drug resistance versus age group

Age group	Odds ratio	Robust standard error	z	P>|z|	[95% confidence interval]	
0-4	2.937896	0.96821	3.27	0.001	1.539981	5.604766
5-9	4.153992	1.61323	3.67	<0.001	1.940421	8.892737
10-14	0.889149	0.163914	-0.64	0.524	0.619522	1.276123
15-17	1	(base)				
_cons	3.460526	0.450915	9.53	0	2.680578	4.467411

b) Model for HIV-1 drug resistance versus drug regimen

Drug regimen	Odds ratio	Robust standard error	z	P>|z|	[95% confidence interval]	
ABC-based	1	(base)				
AZT-based	0.9289162	0.16455	-0.42	0.677	0.656438	1.314497
TDF-based	0.7651992	0.369969	-0.55	0.58	0.296636	1.973898
Unknown	1.377358	0.527428	0.84	0.403	0.650277	2.917395
_cons	4.356164	0.56566	11.33	0	3.377329	5.61869

c) Model for HIV-1 drug resistance versus viral load

Viral load (log_{10})	Odds ratio	Robust standard error	z	P>|z|	[95% confidence interval]	
Viral load (log_{10})	0.8403331	0.0770897	-1.9	0.058	0.7020446	1.005862
_cons	9.757249	4.400479	5.05	0	4.031244	23.61651

To evaluate the associations between HIVDR and patient GRT variables, we analysed continuous variables (viral load) and numerically converted categorical variables (age group and drug regimen) using logistic regression and estimated odds ratios with cluster-robust standard errors.

Supplementary Table S2. HIV-1 drug resistance mutations detected in genotypic resistance tests from children and adolescents in KZN, South Africa

PI (N=740)		NRTI (N=740)		NNRTI (N=740)		INSTI (N=7[a])	
**Mutation**	**No. GRTs (%)**	**Mutation**	**No. GRTs (%)**	**Mutation**	**No. GRTs (%)**	**Mutation**	**No. GRTs (%)**
L10F	86; (11.62)	M41L	64; (8.65)	*V90I*	1; (0.14)	*M50I*	1; (14.29)
L10I	19; (2.57)	A62V	6; (0.81)	*A98G*	41; (5.54)	*H51Y*	1; (14.29)
L10R	2; (0.27)	K65E	0; (0.00)	L100I	21; (2.84)	T66A*	0; (0.00)
L10V	5; (0.68)	K65N	0; (0.00)	K101E	31; (4.19)	T66I	1; (14.29)
V11I	1; (0.14)	K65R	10; (1.35)	K101H	11; (1.49)	T66K*	0; (0.00)
K20M	7; (0.95)	*D67E*	1; (0.14)	K101P	11; (1.49)	*L74M*	0; (0.00)
K20R	52; (7.03)	*D67G*	14; (1.89)	K103N	332; (44.86)	*E92G*	0; (0.00)
K20T	18; (2.43)	D67N	77; (10.41)	K103S	27; (3.65)	E92Q	0; (0.00)
L23I	7; (0.95)	*T69del*	1; (0.14)	V106A	0; (0.00)	*T97A*	1; (14.29)
L24I	36; (4.86)	T69ins	0; (0.00)	*V106I*	11; (1.49)	G118R	1; (14.29)
D30N	0; (0.00)	K70E	10; (1.35)	V106M	102; (13.78)	F121Y	0; (0.00)
V32I	9; (1.22)	K70R	58; (7.84)	*V106T*	1; (0.14)	E138A*	0; (0.00)
L33F	31; (4.19)	*K70Q*	3; (0.41)	V108I	17; (2.3)	E138K*	1; (14.29)
M36I	0; (0.00)	*L74I*	23; (3.11)	_E138A_**	90; (12.16)	E138T*	0; (0.00)
M36L	0; (0.00)	L74V	99; (13.38)	_E138G_**	21; (2.84)	G140A*	0; (0.00)
M36V	0; (0.00)	*V75A*	1; (0.14)	_E138K_**	7; (0.95)	G140C*	0; (0.00)
K43T	14; (1.89)	V75I	7; (0.95)	_E138Q_**	6; (0.81)	G140R	0; (0.00)
M46I	124; (16.76)	*V75M*	6; (0.81)	_E138R_**	0; (0.00)	G140S*	0; (0.00)
M46L	9; (1.22)	*V75T*	3; (0.41)	*V179D*	58; (7.84)	Y143C	0; (0.00)
I47A	8; (1.08)	*V75S*	0; (0.00)	*V179F*	0; (0.00)	Y143H	0; (0.00)
I47V	5; (0.68)	F77L	5; (0.68)	_V179L_**	0; (0.00)	Y143R	1; (14.29)
G48M	0; (0.00)	Y115F	88; (11.89)	*V179T*	3; (0.41)	*Y143S*	0; (0.00)
G48V	2; (0.27)	F116Y	0; (0.00)	Y181C	40; (5.41)	S147G	0; (0.00)
I50L	2; (0.27)	Q151M	1; (0.14)	Y181I	0; (0.00)	Q148H	0; (0.00)
I50V	9; (1.22)	M184V	491; (66.35)	Y181V	1; (0.14)	Q148K	0; (0.00)
F53L	5; (0.68)	M184I	1; (0.14)	Y188C	4; (0.54)	Q148R	0; (0.00)
F53Y	0; (0.00)	L210W	11; (1.49)	Y188H	1; (0.14)	*S153F*	0; (0.00)
I54A	0; (0.00)	*T215C*	3; (0.41)	Y188L	48; (6.49)	*S153Y*	0; (0.00)
I54L	2; (0.27)	*T215D*	1; (0.14)	G190A	67; (9.05)	N155H	0; (0.00)
I54M	0; (0.00)	*T215E*	0; (0.00)	*G190E*	7; (0.95)	*S230R*	0; (0.00)
I54S	2; (0.27)	T215F	41; (5.54)	G190S	4; (0.54)	R263K	0; (0.00)
I54T	0; (0.00)	*T215I*	12; (1.62)	_H221Y_**	29; (3.92)		
I54V	141; (19.05)	*T215S*	2; (0.27)	P225H	84; (11.35)		
Q58E	38; (5.14)	*T215V*	1; (0.14)	F227C	2; (0.27)		
I62V	0; (0.00)	T215Y	24; (3.24)	*F227I*	0; (0.00)		
H69K	0; (0.00)	K219E	31; (4.19)	F227L	34; (4.59)		
H69R	0; (0.00)	*K219N*	2; (0.27)	*F227R*	0; (0.00)		
A71T	1; (0.14)	K219Q	31; (4.19)	*F227V*	0; (0.00)		
A71V	20; (2.7)	*K219R*	8; (1.08)	M230I	0; (0.00)		
G73A	0; (0.00)			M230L	8; (1.08)		
G73C	0; (0.00)			*L234I*	1; (0.14)		
G73S	3; (0.41)			_Y318F_***	0; (0.00)		
G73T	0; (0.00)						
T74P	6; (0.81)						
L76V	73; (9.86)						
V77I	0; (0.00)						
V82A	137; (18.51)						
V82C	6; (0.81)						
V82F	2; (0.27)						
V82L	0; (0.00)						
V82M	1; (0.14)						
V82S	1; (0.14)						
V82T	0; (0.00)						
N83D	3; (0.41)						
I84A	0; (0.00)						
I84C	0; (0.00)						
I84V	14; (1.89)						
I85V	0; (0.00)						
N88D	1; (0.14)						
N88S	1; (0.14)						
L89I	3; (0.41)						
L89M	3; (0.41)						
L89V	2; (0.27)						
L90M	14; (1.89)						

HIV-1, human immunodeficiency virus type 1; GRT, genotypic resistance test; INSTI, integrase strand transfer inhibitor; KZN, KwaZulu-Natal province; NNRTI, non-nucleoside reverse transcriptase inhibitor; No., Number; NRTI, nucleoside reverse transcriptase inhibitor; PI, protease inhibitor

Italics indicate accessory or other non-major mutations as classified by the 2022 edition of the IAS–USA drug resistance mutations list in conjunction with Stanford HIV Drug Resistance Database.

[a]Only 7 out of 740 GRTs with resistance had INSTI testing done in addition to PI, NRTI and NNRTI testing.

*Classified as a minor mutation in the 2022 edition of the IAS–USA drug resistance mutations list, but as a major mutation in Stanford HIV Drug Resistance Database

**Classified as a major mutation to rilpivirine only, in the 2022 edition IAS–USA drug resistance mutations list

***Classified as a major mutation to doravirine only, in the 2022 edition IAS–USA drug resistance mutations list

Supplementary Table S3. Antiretroviral drug regimens with genotypic susceptibility scores for children and adolescents from public sector healthcare facilities in KwaZulu-Natal, South Africa

**ART Regimen (with XTC)[a]**	**All GRTs**	**No HIVDR**	**HIVDR**	**Genotypic susceptibility score[b]**		
				**<2**	**2**	**>2**
	**N=851 (100.00%)**	**N=165 (19.39%)**	**N=686 (80.61%)**	**N=335 (39.37%)**	**N=124 (14.57%)**	**N=392 (46.06%)**
**ABC-based**	**391 (45.95%)**	**73 (18.67%)**	**318 (81.33%)**	**228 (58.31%)**	**2 (0.51%)**	**161 (41.18%)**
ABC+AZT+PI/r[c]	23 (2.70%)	9 (39.13%)	14 (60.87%)	3 (13.04%)	0 (0.00%)	20 (86.96%)
ABC+EFV	28 (3.29%)	5 (17.86%)	23 (82.14%)	21 (75.00%)	1 (3.57%)	6 (21.43%)
ABC+PI/r	339 (39.84%)	59 (17.40%)	280 (82.60%)	203 (59.88%)	1 (0.29%)	135 (39.82%)
ABC+PI/r+RAL	1 (0.12%)	0 (0.00%)	1 (100.00%)	1 (100.00%)	0 (0.00%)	0 (0.00%)
**AZT-based**	**434 (51.00%)**	**86 (19.82%)**	**348 (80.18%)**	**97 (22.35%)**	**121 (27.88%)**	**216 (49.77%)**
AZT+DRV/r	1 (0.12%)	0 (0.00%)	1 (100.00%)	1 (100.00%)	0 (0.00%)	0 (0.00%)
AZT+DRV/r+RAL	1 (0.12%)	0 (0.00%)	1 (100.00%)	1 (100.00%)	0 (0.00%)	0 (0.00%)
AZT+DTG	4 (0.47%)	1 (25.00%)	3 (75.00%)	1 (25.00%)	1 (25.00%)	2 (50.00%)
AZT+EFV	8 (0.94%)	0 (0.00%)	8 (100.00%)	7 (87.5%)	1 (12.5%)	0 (0.00%)
AZT+PI/r	419 (49.24%)	85 (20.29%)	334 (79.71%)	87 (20.76%)	119 (28.4%)	213 (50.84%)
AZT+TDF+PI/r	1 (0.12%)	0 (0.00%)	1 (100.00%)	0 (0.00%)	0 (0.00%)	1 (100.00%)
**TDF-based**	**26 (3.06%)**	**6 (23.08%)**	**20 (76.92%)**	**10 (38.46%)**	**1 (3.85%)**	**15 (57.69%)**
TDF+DRV/r+DTG	1 (0.12%)	0 (0.00%)	1 (100.00%)	0 (0.00%)	0 (0.00%)	1 (100.00%)
TDF+DTG	4 (0.47%)	2 (50.00%)	2 (50.00%)	0 (0.00%)	1 (25.00%)	3 (75.00%)
TDF+EFV	2 (0.24%)	0 (0.00%)	2 (100.00%)	2 (100.00%)	0 (0.00%)	0 (0.00%)
TDF+PI/r	19 (2.23%)	4 (21.05%)	15 (78.95%)	8 (42.11%)	0 (0.00%)	11 (57.89%)

ABC, abacavir; ART, antiretroviral therapy; AZT, zidovudine; DRV/r, ritonavir-boosted darunavir; DTG, dolutegravir; EFV, efavirenz; GRT, genotypic resistance test; HIVDR, human immunodeficiency virus type 1 drug resistance; PI/r, ritonavir-boosted protease inhibitor; RAL, raltegravir; TDF, tenofovir disoproxil fumarate; XTC, emtricitabine/lamivudine

[a]All regimens include XTC which is either emtricitabine or lamivudine. PI/r refers to ritonavir-boosted atazanavir or lopinavir and the drug with the lower penalty score in Stanford University HIV Drug Resistance Database was used. Dolutegravir is assumed to have an individual genotypic susceptibility score of 1 in three GRTs where HIV-1 *integrase* testing was not done. GRTs were done at time of antiretroviral treatment failure.

[b]Genotypic susceptibility score calculated for 851 GRTs with complete ART regimen data.

[c]Prescribed regimen not used as standard of care in South African treatment guidelines.

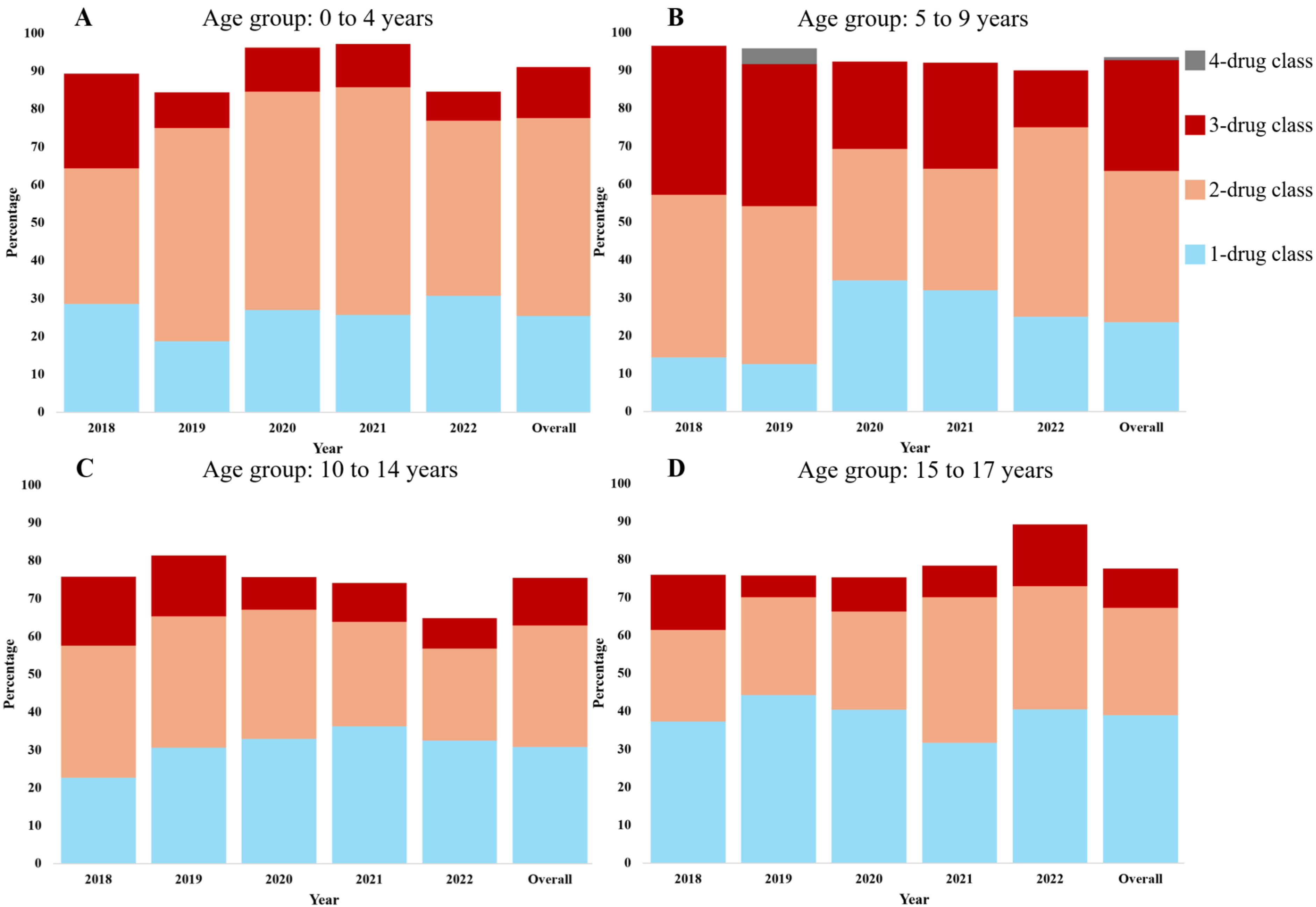

Supplementary Figure S1. (A-D) Temporal changes in the proportions of drug-class resistance observed in children and adolescents from KwaZulu-Natal, South Africa. (B) The child with four-drug class resistance was on a regimen containing zidovudine, lamivudine, ritonavir-boosted darunavir and raltegravir.
