# Supplementary material for: Geospatial analysis reveals similar HIV-1 drug resistance patterns among antiretroviral therapy-experienced children and adolescents (0-17 years) of South Africa
Source: Commun Med (Lond). 2026 Jul 7;6:570. doi: 10.1038/s43856-026-01770-9 (PMC13720034; doi:10.1038/s43856-026-01770-9)
Supplement: Supplementary file 3 [file 43856_2026_1770_MOESM3_ESM.pdf]

Description of Additional Supplementary Files

File name: Supplementary Data 1

Description: Source Data for all time-intensity curves
